# Population-level impact of an accelerated HIV response plan to reach the UNAIDS 90-90-90 target in Côte d’Ivoire: Insights from mathematical modeling

**DOI:** 10.1371/journal.pmed.1002321

**Published:** 2017-06-15

**Authors:** Mathieu Maheu-Giroux, Juan F. Vesga, Souleymane Diabaté, Michel Alary, Stefan Baral, Daouda Diouf, Kouamé Abo, Marie-Claude Boily

**Affiliations:** 1 Department of Epidemiology, Biostatistics, and Occupational Health, McGill University, Montréal, Québec, Canada; 2 Department of Infectious Disease Epidemiology, Imperial College London, St Mary’s Hospital, London, United Kingdom; 3 Centre de recherche du CHU de Québec - Université Laval, Québec, Canada; 4 Département d’infectiologie et santé publique, Université Alassane Ouattara, Bouaké, Côte d’Ivoire; 5 Département de médecine sociale et préventive, Université Laval, Québec, Canada; 6 Institut national de santé publique du Québec, Québec, Canada; 7 Key Populations Program, Department of Epidemiology, Johns Hopkins Bloomberg School of Public Health, Baltimore, Maryland, United States of America; 8 Enda Santé, Dakar, Sénégal; 9 Programme National de Lutte contre le SIDA, Ministère de la Santé et de l’Hygiène Publique, Abidjan, Côte d’Ivoire; University of Bern, SWITZERLAND

## Abstract

**Background:**

National responses will need to be markedly accelerated to achieve the ambitious target of the Joint United Nations Programme on HIV/AIDS (UNAIDS). This target aims for 90% of HIV-positive individuals to be aware of their status, for 90% of those aware to receive antiretroviral therapy (ART), and for 90% of those on treatment to have a suppressed viral load by 2020, with each individual target reaching 95% by 2030. We aimed to estimate the impact of various treatment-as-prevention scenarios in Côte d’Ivoire, one of the countries with the highest HIV incidence in West Africa, with unmet HIV prevention and treatment needs, and where key populations are important to the broader HIV epidemic.

**Methods and findings:**

An age-stratified dynamic model was developed and calibrated to epidemiological and programmatic data using a Bayesian framework. The model represents sexual and vertical HIV transmission in the general population, female sex workers (FSW), and men who have sex with men (MSM). We estimated the impact of scaling up interventions to reach the UNAIDS targets, as well as the impact of 8 other scenarios, on HIV transmission in adults and children, compared to our baseline scenario that maintains 2015 rates of testing, ART initiation, ART discontinuation, treatment failure, and levels of condom use. In 2015, we estimated that 52% (95% credible intervals: 46%–58%) of HIV-positive individuals were aware of their status, 72% (57%–82%) of those aware were on ART, and 77% (74%–79%) of those on ART were virologically suppressed. Reaching the UNAIDS targets on time would avert 50% (42%–60%) of new HIV infections over 2015–2030 compared to 30% (25%–36%) if the 90-90-90 target is reached in 2025. Attaining the UNAIDS targets in FSW, their clients, and MSM (but not in the rest of the population) would avert a similar fraction of new infections (30%; 21%–39%). A 25-percentage-point drop in condom use from the 2015 levels among FSW and MSM would reduce the impact of reaching the UNAIDS targets, with 38% (26%–51%) of infections averted. The study’s main limitation is that homogenous spatial coverage of interventions was assumed, and future lines of inquiry should examine how geographical prioritization could affect HIV transmission.

**Conclusions:**

Maximizing the impact of the UNAIDS targets will require rapid scale-up of interventions, particularly testing, ART initiation, and limiting ART discontinuation. Reaching clients of FSW, as well as key populations, can efficiently reduce transmission. Sustaining the high condom-use levels among key populations should remain an important prevention pillar.

## Abstract 2

Mathieu Maheu-Giroux and colleagues model several potential scenarios for reaching HIV prevention targets in Côte d'Ivoire and estimate the fraction of new infections prevented and changes incidence from 2015–2030.

## Author summary

**Why was this study done?:**

- The 90-90-90 objective of the Joint United Nations Programme on HIV/AIDS (UNAIDS) aims to substantially scale up antiretroviral therapy such that by 2020, 90% of people living with HIV (PLWH) will know their status, 90% of those knowing their status will receive antiretroviral therapy (ART), and 90% of those on ART will have a suppressed viral load (with coverage increasing to 95-95-95 by 2030).
- Our study examines the impact of reaching, and missing, the UNAIDS targets in different populations (female sex workers, clients of female sex workers, men who have sex with men, and the general heterosexual population) and assesses the influence of condom use among key populations in Côte d’Ivoire.
- Côte d’Ivoire is an interesting case study because it has one of the highest estimated HIV incidences in West Africa and a high prevalence in the general population (3.7% in 2012) and because key populations are important to overall HIV transmission.

**What did the researchers do and find?:**

- Using a transmission dynamic mathematical model, we simulated the HIV epidemic in Côte d’Ivoire to estimate the fraction of new HIV infections that could be prevented under different scenarios, and we estimated changes in HIV incidence between 2015 and 2030.
- Reaching the universal UNAIDS targets in time would halve the number of new HIV infections over 2015–2030, compared to our baseline scenario (prevented fraction of 50%). If the 90-90-90 objective is reached with a 5-year delay in 2025—instead of 2020—the fraction of infections averted would be smaller (prevented fraction of 30%).
- Attaining the UNAIDS objectives only in men who have sex with men and female sex workers and their clients would provide an impact equivalent to that of missing the 90-90-90 objective by 5 years (prevented fraction of 30%).

**What do these findings mean?:**

- Substantial reductions in the number of new HIV infections could be obtained in Côte d’Ivoire with rapid treatment scale-up. The programmatic efforts required are nevertheless important and need the strengthening of HIV testing and the whole care continuum.
- In settings like Côte d’Ivoire, where core group interventions have been key to HIV prevention, sustaining high condom use among key populations should be emphasized in prevention programs so as to not jeopardize the impact of reaching the UNAIDS targets.
- Finally, considering clients of female sex workers, as well as female sex workers and men who have sex with men, as part of the UNAIDS 90-90-90 response is important to efficiently reduce population-level HIV transmission.

## Introduction

Despite a long-standing national HIV response, HIV incidence in Côte d’Ivoire is the highest in West Africa [1]. Past interventions, especially promotion and distribution of condoms among female sex workers (FSW), have been effective at averting infections, but low coverage of antiretroviral therapy (ART) and prevention of mother-to-child transmission (PMTCT) resulted in suboptimal population-level impact during the last decade [2]. As with many countries, the national response in Côte d’Ivoire will need to be markedly accelerated in order to reach the ambitious 90-90-90 objective of UNAIDS [3]. The UNAIDS target specifies that, by 2020, 90% of people living with HIV (PLWH) will be aware of their status, 90% of diagnosed PLWH will receive ART, and that 90% of those on treatment will be virologically suppressed (with 95-95-95 coverage by 2030) [4,5]. Current estimates for these indicators point to gaps in the response [3]. Achieving high treatment coverage is nevertheless possible in sub-Saharan Africa, as demonstrated by Botswana’s experience [6].

UNAIDS mathematical modeling predictions suggest that reaching the 90-90-90 objective by 2020—and attaining 95-95-95 coverage by 2030—would reduce the number of new HIV infections worldwide by nearly 90% in 2030, as compared to maintaining 2013 intervention coverage levels [5]. Such reductions in incidence would contribute to “end the AIDS epidemic as a major global health threat” [4]. The impact of fast tracking the response is context specific [7], however, and is likely to also hinge on reaching the same target coverage in key populations. In addition to the 3 coverage objectives, UNAIDS also emphasized the scale-up of HIV prevention, such as condom use. It is nevertheless expected that about 60% of the projected decline in new infections will be attributed to ART [5].

HIV prevalence in Côte d’Ivoire declined to 3.7% in 2011–2012 [8] but remains substantially higher among MSM and FSW [9,10], who are identified as key populations in the country’s most recent national strategic plan [11]. Yet, Côte d’Ivoire’s emphasis on ART scale-up has recently resulted in funding declines for condoms and prevention activities for key populations [3]. If this trend is not reversed, achieving substantial reductions in new infections could be jeopardized. That is because, even if the 90-90-90 objective was reached by 2020, 27% of infected individuals with unsuppressed viral load would still contribute to HIV transmission in the population [5].

To help inform the acceleration of Côte d’Ivoire’s national HIV response, a comprehensive review of epidemiological and programmatic data was completed. A detailed dynamic model of HIV transmission was developed, parameterized, and calibrated to local data [12]. The main objective of our study is to estimate the population-level impact of reaching the UNAIDS 90-90-90 target by 2020 (and 95-95-95 by 2030). Secondary objectives are to examine the impact of 8 other scenarios with different scale-up speeds, coverage achieved in the general and key populations, and condom use in key populations on both adult and pediatric HIV infections.

## Methods

### Transmission model

We developed an age-stratified deterministic model of sexual and vertical HIV transmission, whose detailed description can be found elsewhere [12]. Briefly, the model represents an open and growing population (15–59 years old) stratified in 8 risk groups: low-risk females, high-risk females (>1 partner year^-1^), FSW, low-risk males, high-risk males (>2 partner year^-1^), clients of female sex workers (CFSW), bisexual MSM, and exclusive MSM. Each risk group was further stratified in 4 age classes: 15–19, 20–24, 25–49, and 50–59 years old. To reflect the reported increase in the proportion of those aged 15–19 years who are still virgins over time [8,13–15], we assumed individuals to have their sexual debut either at 15 or at 20 years of age. Individuals leave the modeled population because of aging, HIV-related mortality, or HIV-unrelated mortality.

The annual rate at which susceptible individuals acquire HIV infections (the force of infection) depends on the number and type of sexual partners, HIV prevalence among partners, sexual mixing patterns between age/risk groups, the type of sex act (vaginal and insertive/receptive anal intercourse), the fraction of sex acts protected by condoms, the partner’s infectiousness (i.e., varying by disease stage, ART treatment, and viral suppression status), and the uninfected partner’s susceptibility (e.g., young women have a higher risk of acquiring HIV [16,17]). The protective effect of male circumcision on HIV acquisition risk was not modeled per se but captured in the per-act HIV transmission probability since more than 95% of men in Côte d’Ivoire are circumcised [8,14].

Following infection, untreated HIV-positive individuals progress through a short highly infectious primary infection, followed by 4 disease stages, defined based on CD4 cell counts (the model’s flowchart is presented in Maheu-Giroux et al. [12]). All individuals in these 4 stages can be tested for HIV at a rate that depends on calendar time, presence of symptoms, and the risk group to which they belong. Once diagnosed, positive individuals initiate treatment at a per capita rate that also depends on calendar time and CD4 count levels. Treated individuals have lower rates of HIV-related mortality than those not receiving ART. Viral suppression is achieved after a short time lag following treatment initiation. Finally, some individuals may experience a therapeutic failure or discontinue ART, in which case the disease follows its natural progression unless these individuals initiate treatment again.

Mother-to-child transmission (MTCT) of HIV was modeled using a linked decision tree model to predict the number of infants born to HIV-positive mothers who would be vertically infected (described in Maheu-Giroux et al. [12]). Age-specific fertility rates are applied to the population of HIV-positive women derived from the deterministic model. Depending on the availability and type of PMTCT interventions available, pregnant women can be already on ART, not tested or not attending antenatal care, or tested during antenatal care visits. When tested, pregnant women can either be given ART prophylaxis, initiate ART treatment, or do nothing. In each case, the probability of MTCT depends on the breastfeeding status of the infant and the mother’s CD4 cell count. Women tested and/or receiving ART during their pregnancy are then reallocated to the corresponding compartment of the dynamic model.

### Model parameterization

A comprehensive review of scientific and grey literatures, complemented with on-site meetings with program managers, was performed to inform model parameters and intervention coverage. Demographical parameters came from the UN’s World Population Prospects (Table A in S1 Appendix) [18]. The country’s 3 Demographic and Health Surveys and its AIDS Indicator Survey [8,13–15] informed sexual behaviors of the general heterosexual population (Table B in S1 Appendix). Biological parameters, those for PMTCT, and the sexual behaviors of MSM and FSW were abstracted from the scientific literature (Tables B–D in S1 Appendix). Data on historical trends of past interventions, such as condom use, treatment, and PMTCT, were abstracted from government reports and the scientific literature (Tables E–H in S1 Appendix). Further information on sexual mixing, the model’s equations, and equations for the force of HIV infection and for PMTCT, as well as details on the estimation of past trends for condom use, HIV testing, and PMTCT and ART coverage, can be found elsewhere [12].

### Model calibration

Calibration of the mathematical model entails using statistical techniques to select combinations of parameter sets that best reproduce temporal trends in epidemiological data. This was achieved by first eliciting prior distributions to capture parameters’ uncertainty. A Bayesian melding approach was adopted [19,20], and incremental mixture importance sampling (IMIS) [21] was then used to efficiently sample posterior distributions. IMIS’s initial sampling stage consisted of Latin hypercube sampling of 5 million parameter sets. Sets producing model predictions within prespecified constraints for the following outcomes were accepted: national HIV prevalence by sex and age groups [8,14,22], HIV prevalence among FSW [9,23–26], HIV prevalence among MSM by age [10], HIV prevalence among CFSW [27], and overall ART coverage from 2002 to 2014 [28–34]. Binomial likelihoods for HIV prevalence data were calculated by age group for both men and women in the general population and for FSW, CFSW, and MSM (by age group for the latter). The model’s likelihood was calculated by summing the binomial log-likelihoods of all those preceding outcomes. Model outputs that did not fall within the prespecified constraints were assigned a likelihood of zero (ART coverage was only included in the likelihood as prespecified constraints). The data used for model calibration are presented elsewhere [12]. The model’s posterior distributions are summarized using medians and 95% credible intervals.

The model, coded in MATLAB, was initialized in 1970, and HIV was seeded in FSW, CFSW, and MSM in 1975. It was solved numerically using a Euler algorithm with a 0.05-year time step.

### Scenarios and analyses

We assessed the population-level impact of 10 different intervention scenarios starting in 2015, the year of the last country assessment (Table 1). The baseline scenario (SC1) assumes that rates of HIV testing, ART initiation, ART discontinuation, and therapeutic failure, as well as PMTCT coverage and condom use levels, are maintained at their 2015 values through 2030. Scenario 2 (SC2) assumes that rates of HIV testing and ART recruitment continue to increase over the 2015–2020 period at the same rate as during 2010–2015 and remain constant after 2020. Scenario 3 (SC3) corresponds to reaching the UNAIDS 90-90-90 and 95-95-95 objectives in 2020 and 2030, respectively. In scenario 4 (SC4), the 90-90-90 targets are reached with a 5-year delay, in 2025, and this coverage remains constant through 2030. Scenario 5 (SC5) is similar to SC3, but the 95-95-95 coverage is not achieved in 2030. Scenario 6 (SC6) assumes that UNAIDS objectives are reached only in the general population, while key populations continue to experience 2015 testing, treatment, ART failure, and ART discontinuation rates (as in SC1). In contrast, scenario 7 (SC7) simulates that UNAIDS objectives are reached in key populations only, and not in the general population, who continue to experience 2015 testing, treatment, failure, and ART discontinuation rates (as in SC1). Scenario 8 (S8) is a modification of SC7, in which the UNAIDS objectives are additionally reached in CFSW. Two additional scenarios assess the impact of a change in condom use among key populations. Scenario 9 (SC9) is the same as SC3, but the proportion of condom-protected sex acts increases linearly from their observed 2015 levels to 95% in 2020 for both FSW and MSM. In contrast, the proportion of condom-protected sex acts among FSW and MSM decreases linearly by 25 percentage points (from their 2015 levels) between 2015 and 2020 for scenario 10 (SC10).

**Table 1:** Description of different intervention scenarios considered in Côte d’Ivoire over the 2015–2030 time period.

Scenarios	HIV testing, ART initiation, and ART failure rates	ART discontinuation rates	PMTCT	Condom use (varies by risk and age groups)
Baseline (SC1)	Stable at 2015 rates.	Stable at 2015 rates.	Stable at 2015 coverage.	Stable at 2015 coverage*.
Current trends (SC2)	Increasing trends in rates observed during 2010–2015 extrapolated over 2015–2020 with the rates stable afterwards.	As SC1.	Trends in observed rates during 2010–2015 extrapolated over 2015–2020 (with the rates stable afterward).	As SC1.
UNAIDS (SC3)	Rates linearly modified to yield 90-90-90 coverage by 2020 and 95-95-95 coverage by 2030.	ART discontinuation linearly decreases to a rate of 1/30 year^-1^ by 2020 and to 1/40 year^-1^ by 2030.	95% of pregnant women tested during ANC visits by 2020. All pregnant women testing positive are given ART by 2020.	As SC1.
Delayed UNAIDS (SC4)	Rates linearly modified to yield 90-90-90 coverage by 2025 and stable afterward.	ART discontinuation linearly decreases to a rate of 1/30 year^-1^ by 2025 and is stable afterward.	As SC3.	As SC1.
UNAIDS 90-90-90 to 2030 (SC5)	Rates linearly modified to yield 90-90-90 coverage by 2020 and stable afterward.	ART discontinuation linearly decreases to a rate of 1/30 year^-1^ by 2020 and is stable afterward.	As SC3.	As SC1.
UNAIDS in general population (SC6)	For the general population only: as SC3. FSW and MSM experience 2015 rates.	As SC3 for the general population. FSW and MSM experience 2015 rates of ART discontinuation.	As SC3.	As SC1.
UNAIDS in KP (SC7)	As SC1 for the general population. For MSM and FSW: rates modified to yield 90-90-90 coverage by 2020 and 95-95-95 coverage by 2030.	As SC3 for MSM and FSW. The general population experience 2015 rates of ART discontinuation.	As SC1	As SC1.
UNAIDS in KP and CFSW (SC8)	As SC1 for the general population. For MSM, FSW, and CFSW: rates modified to yield 90-90-90 coverage by 2020 and 95-95-95 coverage by 2030.	As SC3 for MSM, FSW, and CFSW. The general population experience 2015 rates of ART discontinuation.	As SC1.	As SC1.
UNAIDS with condom use increases (SC9)	As SC3.	As SC3.	As SC3.	Linear increase from 2015 to 95% of sex acts protected for FSW and MSM by 2020 (stable afterward).
UNAIDS with condom use decreases (SC10)	As SC3.	As SC3.	As SC3.	Linear drop in condom use by 25 percentage points from 2015 levels to 2020 for FSW and MSM (stable afterward).

ANC, antenatal care; ART, antiretroviral therapy; CFSW, client(s) of female sex workers; FSW, female sex worker(s); KP, key population(s); MSM, men who have sex with men; PMTCT, prevention of mother-to-child transmission; SC, scenario; UNAIDS, Joint United Nations Programme on HIV/AIDS.

*In 2015, we estimated that the proportion of sex acts protected by a condom was 24% (95% credible intervals [95% CrI] 17%–32%, weighted average of age-specific proportions) in the general population, 87% (95% CrI 84%–91%) during sex work between FSW and CFSW, and 73% (95% CrI 64%–80%) during MSM sexual contacts.

UNAIDS coverage targets were implemented by sequentially optimizing the HIV testing rate, ART initiation rate, and ART failure rate to reach the first, second, and third UNAIDS targets, respectively, at the specified time in the relevant groups. To reflect logistical constraints, we assumed that uninfected or asymptomatic individuals in the general population cannot experience testing rates above once per year (FSW and MSM can test up to 4 times per year). Strengthening of the care continuum is an important step toward reaching high levels of viral suppression, and loss to follow-up could compromise this objective. In this study, we only considered ART discontinuation, one of the components of loss to follow-up, as the parameter influencing retention in care. For all scenarios in which the UNAIDS objective is reached, rates of ART discontinuation were assumed to linearly decrease from their 2015 levels to 1/30 year^-1^ in 2020 (2025 for SC4 and constant after for SC5) and to 1/40 year^-1^ in 2030. Low rates of ART discontinuation were assumed because, normatively, individual-level and population-level benefits of ART are maximized when HIV-positive individuals do not discontinue treatment [35,36]. For SC1 and SC2, the rates of ART discontinuation were assumed to remain at their 2015 values. Regarding PMTCT, the proportion of HIV-positive pregnant women accessing antenatal care (ANC) clinics and being tested for HIV was assumed to reach a maximum of 95% in scenarios in which the UNAIDS targets are reached [37]. Further, the proportion of pregnant women testing positive who receive lifelong ART gradually increases to 100% at the time the UNAIDS target is reached. Because it would be impractical to target PMTCT activities to FSW only, SC7 and SC8 did not differ regarding PMTCT between FSW and women not engaging in commercial sex. Apart from SC9 and SC10, which explore changes in the fraction of sex acts protected by condom among FSW and MSM, all other scenarios assume that condom use remains at the 2015 levels.

For each scenario, we predicted HIV prevalence and annual numbers of new HIV infections (for adults and children) and HIV-related deaths for the 2015–2030 period. To assess the population-level impact of the different scenarios, the fraction of HIV infections prevented (PF) over 2015–2030 was estimated by comparing the cumulative number of incident infections between each specific scenario and the baseline SC1. Prevented fractions were calculated for sexually and vertically transmitted infections separately. Sensitivity analyses were also performed by exploring how the fitted parameter values influence prevented fraction estimates.

## Results

The calibrated model produced estimates of HIV prevalence and ART coverage that corresponded to the empirical ones from the different epidemiological surveys (Fig A in S1 Appendix) and programmatic data available [12]. Estimates of incidence, prevalence, and AIDS mortality were also consistent with UNAIDS estimates (Table I in S1 Appendix).

### HIV epidemiology under the baseline scenario (SC1)

**Fig 1:**
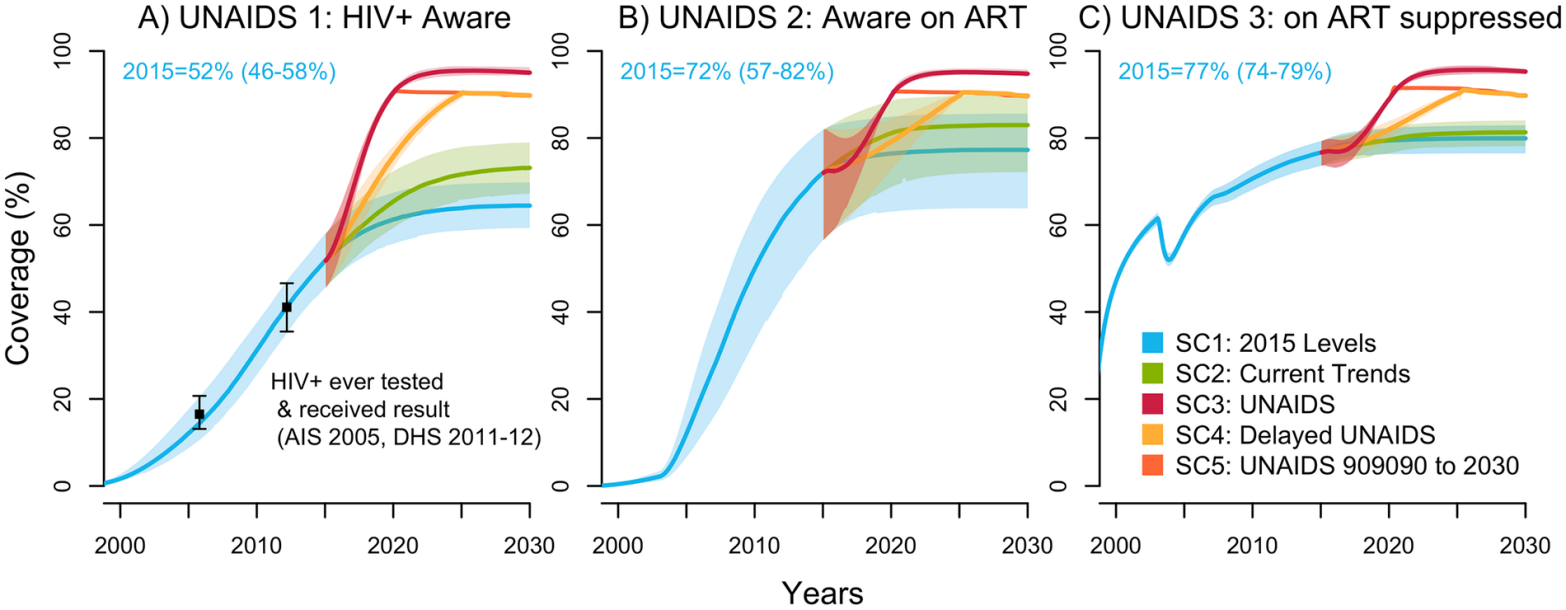
UNAIDS indicators under selected intervention scenarios among the population aged 15–59 years in Côte d’Ivoire (median with 95% credible intervals). The UNAIDS indicators from left to right are (A) proportion of HIV-positive individuals aware of their status (data from [8,14], used for cross-validation), (B) proportion of those aware who are receiving antiretroviral therapy (ART) (the model was fitted to the proportion of HIV-positive individuals on ART), and (C) proportion of those on ART who are virally suppressed. The scenarios are as defined in Table 1. (SC1) Baseline: testing rate, ART recruitment rate, and ART failure rate stable at their 2015 values; (SC2) observed increase in those 3 rates from 2010–2015 projected through 2020; (SC3) UNAIDS: 90-90-90 objective reached in 2020 and 95-95-95 in 2030; (SC4) delayed UNAIDS: 90-90-90 objective reached in 2025 and maintained to 2030; and (SC5) UNAIDS 90-90-90 to 2030: 90-90-90 objective reached in 2020 and maintained to 2030. AIS, AIDS Indicator Survey; DHS, Demographic Health Survey; SC, scenario; UNAIDS, Joint United Nations Programme on HIV/AIDS.

In 2015, the model estimated that the proportion of PLWH in Côte d’Ivoire aware of their status was 52% (46%–58%), the fraction of those aware on ART was 72% (57%–82%), and the fraction receiving ART who are virologically suppressed was 77% (74%–79%) (Fig 1). In other words, this means that 37% (28%–42%) of all PLWH were estimated to be on ART and 29% (22%–33%) were virologically suppressed in 2015. These indicators are presented for FSW and MSM in Fig B and Fig C in S1 Appendix, respectively.

**Fig 2:**
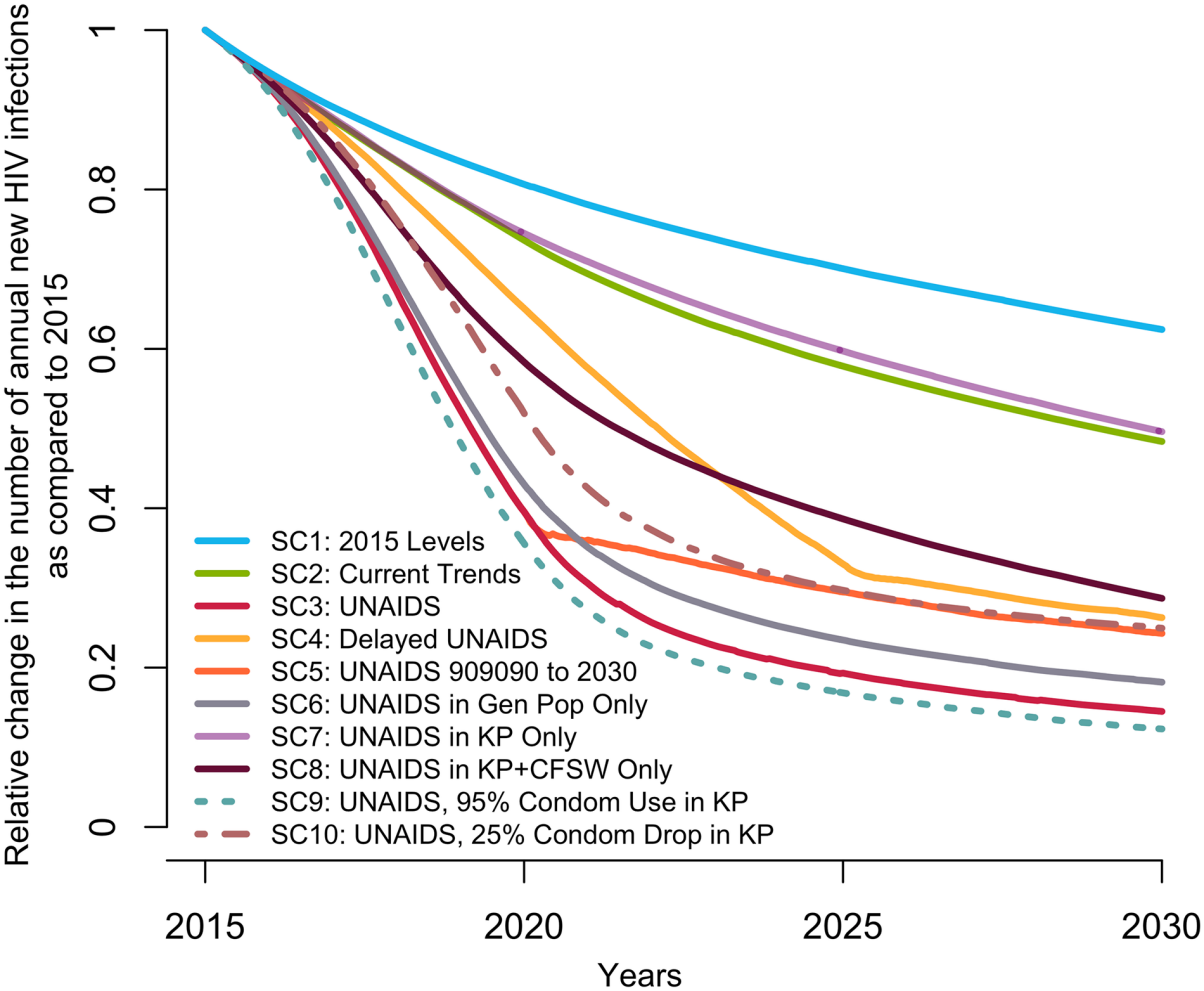
Predicted median relative change in annual number of new HIV infections among 15–59-year-olds in Côte d’Ivoire from 2015 to 2030 under different intervention coverage scenarios. The scenarios are detailed in Table 1. (SC1) Baseline: testing rate, antiretroviral therapy (ART) recruitment rate, and ART failure rate stable at their 2015 values; (SC2) current trends: observed increase in those 3 rates from 2010–2015 projected through 2020; (SC3) UNAIDS: 90-90-90 objective reached in 2020 and 95-95-95 in 2030; (SC4) delayed UNAIDS: 90-90-90 objective reached in 2025 and maintained to 2030; (SC5) UNAIDS 90-90-90 to 2030: 90-90-90 objective reached in 2020 and maintained to 2030, (SC6) UNAIDS in general population: 90-90-90 objective reached in 2020 and 95-95-95 in 2025 among the general population only; (SC7) UNAIDS in key populations (KP): 90-90-90 objective reached in 2020 and 95-95-95 in 2030 among FSW and MSM populations only; (SC8) UNAIDS in key populations and CFSW: 90-90-90 objective reached in 2020 and 95-95-95 in 2030 among MSM, FSW, and CFSW only; (SC9) UNAIDS plus condom use increases in key populations: 90-90-90 objective reached in 2020 and 95-95-95 in 2030 and a rise to 95% by 2020 of sexual acts protected by a condom among FSW and MSM; and (SC10) UNAIDS with condom drop in key populations: 90-90-90 objective reached in 2020 and 95-95-95 in 2030 and a decline by 25 percentage points of sexual acts protected by a condom among FSW and MSM. (95% credible intervals are not presented to ease visual interpretation.) CFSW, client(s) of female sex workers; FSW, female sex worker(s); Gen Pop, general population; MSM, men who have sex with men; SC, scenario; UNAIDS, Joint United Nations Programme on HIV/AIDS.

Under the baseline scenario, the number of new HIV infections among 15–59-year-olds is expected to decline by 37% (23%–50%) from 2015 to 2030 given the predicted increase in treatment coverage that would result from maintaining intervention rates constant at their 2015 values (Fig 2). The number of HIV-related deaths would decrease by 39% (26%–49%) for the same period among this age group (Fig D in S1 Appendix), resulting in a 2030 HIV prevalence of 1.3% (0.9%–1.8%) compared to 3.2% (2.5%–3.9%) in 2015 (Fig E in S1 Appendix).

### Impact of the scenario projecting current trends (SC2)

**Fig 3:**
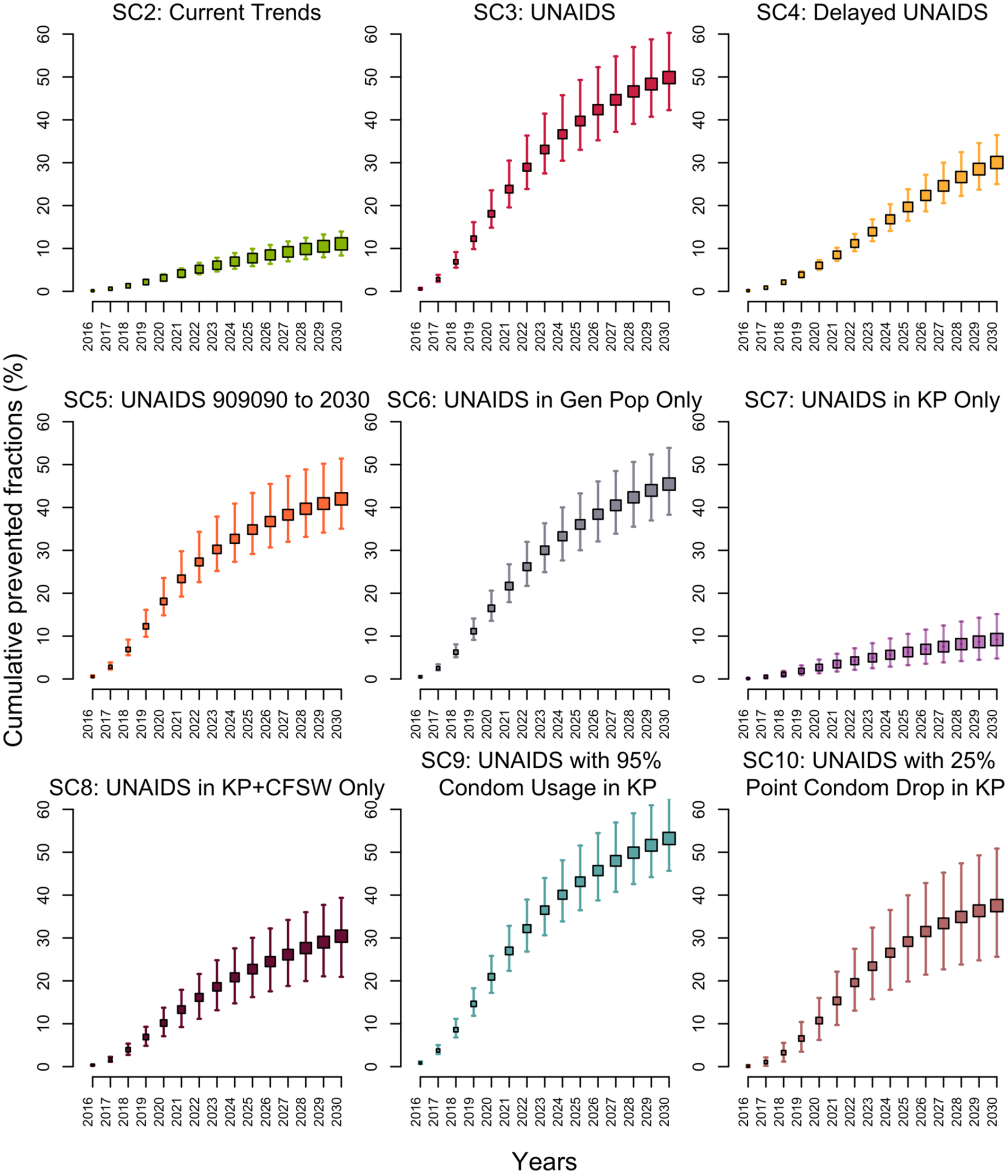
Cumulative fractions of all new HIV infections prevented (medians and 95% credible intervals) among 15–59-year-olds in Côte d’Ivoire between 2015 and 2030 for different intervention scenarios compared to baseline scenario 1 as the counterfactual. (Details of the scenarios can be found in Table 1.) CFSW, client(s) of female sex workers; Gen Pop, general population; KP, key population(s); SC, scenario; UNAIDS, Joint United Nations Programme on HIV/AIDS.

If testing and ART initiation rates continued to increase over the 2015–2020 period as they did over 2010–2015 (SC2), the 3 UNAIDS targets would be missed, reaching only 66% (59%–71%), 83% (71%–91%), and 80% (77%–82%) of PLWH aware, PLWH aware on ART, and PLWH on ART suppressed by 2020, respectively. This translate to 44% (36%–48%) of all PLWH being virologically suppressed. This scenario is estimated to avert 11% (9%–14%) of new HIV infections over 2015–2030 compared to the baseline scenario SC1 (Fig 3).

### Impact of the UNAIDS 90-90-90 (and 95-95-95 in 2030) scenario (SC3)

To reach the UNAIDS targets in 2020, the number of HIV tests performed from 2015 to 2020 needed to increase by a factor of 5 (4 to 7), the total number of ART initiations needed to grow by 20% (7%–57%), and the ART discontinuation rate needed to reduce by a factor of 6 (5 to 8) compared to the baseline scenario (SC1). Although testing frequency must remain high over time, the annual number of ART initiations in the UNAIDS scenario (SC3) is expected to decrease by 75% (70%–80%) over 2020–2030, as compared to 2015–2020, because the widest ART coverage gap is filled in the first time period.

Reaching the UNAIDS targets on time (SC3) would reduce the annual number of new HIV infections by 85% (76%–92%) in 2030 compared to 2015. The expected number of HIV-related deaths would decrease by 66% (59%–72%) in 2030 as compared to 2015, and HIV prevalence would decline to 0.8% (0.6%–1.2%) in 2030. Compared to the baseline scenario (SC1), reaching the UNAIDS targets on time (SC3) would avert 50% (42%–60%) of new HIV infections over 2015–2030 (Fig 3).

### Impact of missing the UNAIDS targets (SC4 and SC5)

Realizing the UNAIDS 90-90-90 objective with a 5-year delay (SC4) would reduce the fraction of HIV infections averted to 30% (25%–36%). In contrast, if the first 90-90-90 objective is reached in 2020 but the coverage of the 3 UNAIDS indicators stays at that level through 2030 (SC5), 42% (35%–51%) of infections would still be averted (Fig 3).

### Impact of coverage levels achieved in different populations (SC6, SC7, and SC8)

If the UNAIDS targets were attained on time in the general population only (including CFSW) and were missed for key populations, the impact would be similar (SC6; PF = 45%; 38%–54%) to the scenario in which the UNAIDS targets are also reached among key populations (SC3; Fig 3). On the other hand, reaching the UNAIDS targets among key populations (SC7) would prevent 20% (13%–30%) of new MSM and FSW HIV infections, leading to the smallest overall fraction of infection averted over 2015–2030 across all scenarios (PF = 9%; 5%–15%). Although this scenario would not avert large fractions of new HIV infections, it is the one that requires reaching the smallest number of people: FSW and MSM constitute 1.4% (1.0%–1.8%) of the total population. If the UNAIDS targets are reached for MSM, FSW, and CFSW (but not in the general population), the effect on overall infections averted would triple (SC8; PF = 30%; 21%–39%) and achieve an impact equivalent to that of missing the UNAIDS 90-90-90 targets by 5 years (SC4).

### Impact of changes in condom use among key populations (SC9 and SC10)

The only scenario outperforming the UNAIDS targets (SC3) was SC9, which assumed that condom use would increase to 95% of sex acts among key populations, with 53% of infections averted (46%–63%) over 2015–2030 (Fig 3). Importantly, reaching the UNAIDS targets, at the cost of a 25-percentage-point decrease in condom use among key populations in 2020 (SC10), would considerably reduce impact, with a prevented fraction of 38% (26%–51%).

### Short-term impact, predicted incidence, and sensitivity analyses

For all scenarios, the population-level impact on infections averted is greatest over the long term, with much smaller prevented fractions over 2015–2020 than over 2015–2030 (Fig 3). For example, the 5-year impact (2015–2020) of the UNAIDS scenario (SC3) is less than half (PF = 18%; 15%–24%) of the impact achieved over 15 years. Interestingly, all scenarios predict that the median incidence among 15–59-year-olds would fall below the proposed elimination threshold of 1 per 1,000 person-years by 2030 (Fig F in S1 Appendix), but this could be achieved faster under the UNAIDS scenario (SC3). Sensitivity analyses suggest that our prevented fraction estimates are sensitive to the assumed ART efficacy to reduce HIV transmission. Condom effectiveness, baseline rates of ART discontinuation, and therapeutic failures did not affect our results (Fig G in S1 Appendix). Finally, we also examined the influence of assuming lower ART discontinuation rates (1/10 year^-1^ in 2020 and 1/15 year^-1^ in 2030) in the main UNAIDS scenarios (SC3) and found that it did not affect our results (PF = 50%; 42%–60%), as this lower treatment retention could be effectively compensated by higher ART reinitiations.

### Impact of the different scenarios on pediatric HIV infections

**Fig 4:**
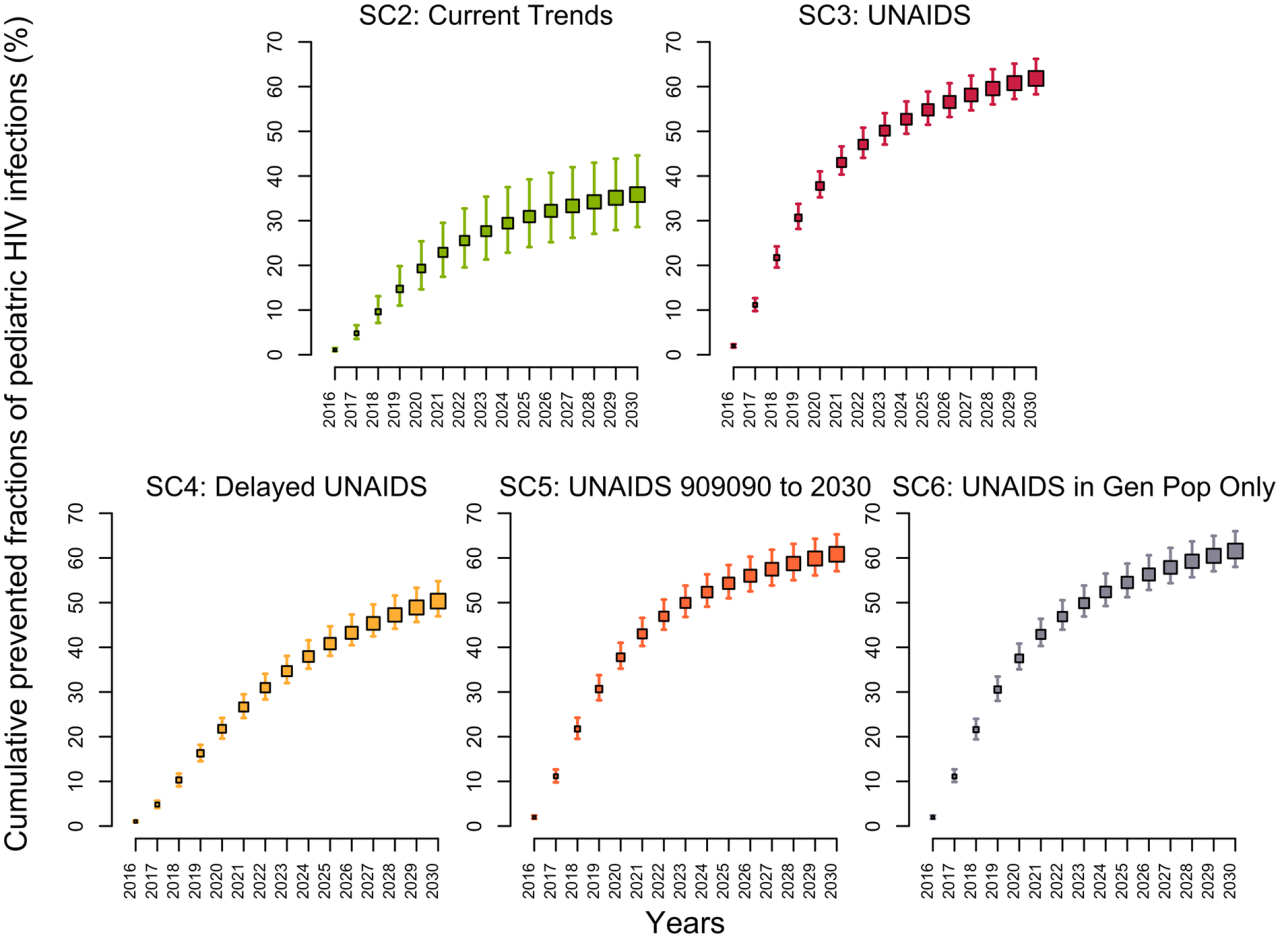
Cumulative fractions of new HIV pediatric infections prevented (medians and 95% credible intervals) in Côte d’Ivoire between 2015 and 2030 for different intervention scenarios using the 2015 intervention coverage levels (scenario 1) as the counterfactual. (Descriptions of the scenarios can be found in Table 1). SC, scenario; UNAIDS, Joint United Nations Programme on HIV/AIDS.

The model estimated that 64% (58%–69%) of HIV-positive pregnant women were receiving either ART prophylaxis or ART treatment in 2015 (Fig H in S1 Appendix). If PMTCT coverage was maintained at its 2015 level (SC1), the fraction of HIV-infected infants born to HIV-positive mothers would decrease from 17% (14%–20%) in 2015 to 14% (12%–17%) in 2030. Under the UNAIDS scenario (SC3), the fraction of infants born to HIV-positive mothers acquiring the virus would decrease below 3.7% (3.6%–3.8%) in 2030, averting 62% (58%–66%) of pediatric infections over 2015–2030 (Fig 4). Reaching the UNAIDS target with a 5-year delay (SC4) would still prevent 50% (47%–55%) of new pediatric HIV infections. In comparison, the second scenario (SC2) assumed that the same rapid rate of increase in PMTCT activities observed between 2010–2015 would be sustained through 2020. This scenario would prevent 36% (29%–45%) of new pediatric HIV infections, resulting in 7% (5%–9%) of infants acquiring HIV from their mothers in 2030.

## Discussion

Using mathematical modeling, we explored the population-level impact of reaching, as well as missing, the UNAIDS targets on new HIV infections averted in Côte d’Ivoire. Our results suggest that reaching the UNAIDS targets on time would prevent 50% of infections in adults and 62% of pediatric infections over 2015–2030, compared to coverage achieved in our baseline scenario. Attaining the 90-90-90 objective with a 5-year delay and maintaining that coverage to 2030 would almost halve the potential impact of this accelerated response. This highlights the importance of rapidly scaling up intervention coverage in order to maximize health gains [37]. In fact, reaching the 90-90-90 objective on time could be more important to achieving short-term impact than reaching the 95-95-95 objective in 2030. Importantly, the population-level impact of the UNAIDS targets will be reduced by a quarter if condom coverage in FSW and MSM decreases by 25%. Given the recent funding cutbacks for condoms and prevention activities for key populations in Côte d’Ivoire [3], consolidating these activities is important. In fact, a model-based evaluation of the HIV response in Côte d’Ivoire suggested that condom use during sex work has been highly effective at preventing transmission in the last decades [2,12].

Efforts needed to achieve the UNAIDS targets cannot be understated [38]. Despite uncertainty in the fraction of PLWH aware of their status, this study points to an important bottleneck in the response: HIV testing. We estimated that the number of tests performed from 2015 to 2020 should be approximately increased by a factor of 5 to reach the first 90 by 2020. As demonstrated in other settings [39,40], this number could be substantially reduced, however, if regions of high prevalence and/or high-risk individuals in the general population are targeted. Routine opt-out HIV testing and counselling at the health facility level could also be considered to address this bottleneck. In terms of ART initiations, the bulk of the efforts to reach the targets needs to occur before 2020, with substantially less initiations needed thereafter. Similarly, retention in treatment needs to rapidly improve, and these improvements need to be sustained after 2020. However, given the uncertainty in empirical estimates of retention rates, quantifying precisely the required improvement is difficult. When optimizing rates to reach the 90-90-90 target, higher discontinuation rates could be compensated by higher reinitiation rates, as our model does not differentiate between new initiations and reinitiations. For example, the same viral suppression targets can be achieved either by reducing ART discontinuation rates from 2015 to 2020 by a factor of 6, as in our UNAIDS scenarios, or by a factor of 2 if we increase reinitiation rates. Nevertheless, strengthening the whole care continuum and taking health systems constraints into consideration [41], from rapid diagnosis to sustained viral suppression [42,43], are required for programmatic success to materialize.

PMTCT coverage has considerably increased between 2010–2015 in Côte d’Ivoire, and projecting this increase over 2015–2020 would decrease the proportion of infants acquiring HIV to 7% in 2030, compared to 17% (14%–20%) in 2015. Reaching the UNAIDS objective would bring this down to 3.7% in 2030. Further reductions could be achieved if replacement feeding was provided and/or if length of breastfeeding was reduced for HIV-positive mothers since such interventions were not modelled. HIV testing and ART initiation due to PMTCT interventions are also important in achieving the UNAIDS targets, with 8% (6%–10%) of total initiations resulting from these PMTCT interventions in 2030.

Our model results are broadly in agreement with those of UNAIDS in terms of a relative decline in HIV infections and deaths [4,5,37]. Direct comparisons are difficult given the differences in baseline levels of interventions and HIV transmission dynamics between Côte d’Ivoire and the countries used to inform the different epidemic types. Monitoring progress towards the UNAIDS targets is challenging. First, obtaining regular data on the proportion of PLWH aware of their status is difficult. It theoretically requires obtaining a nationally representative sample of HIV-positive individuals and, from self-reports, calculating the proportion aware of their status. However, selective underreporting of diagnosed status by HIV-positive individuals from self-reports has been observed in such surveys given the significant stigma related to this infection [44]. Notwithstanding these limitations to monitoring testing progress, UNAIDS recommends using population-based surveys to estimate the proportion of PLWH aware of their status by using the fraction of PLWH reporting having ever been tested and who received their results as the upper bound of this indicator [45]. We cross-validated our results against this indicator and found them to be in accordance with this upper bound (Fig 1), most likely providing a conservative estimate of the HIV testing gap. It is generally easier for national programs to estimate ART coverage since information on the number of PLWH on ART is usually available from facility-based ART registers or drug supply management systems (the denominator can be obtained from population-based serosurveys). Scale-up of viral load monitoring in Côte d’Ivoire is currently too low to empirically estimate levels of viral suppression among PLWH on ART: less than 3% of ART patients had at least 1 viral load test in 2015 [46]. Viral load monitoring using dried blood samples has been deemed cost-effective and could be rapidly expanded to improve ART outcomes and monitor programmatic success [47]. In all cases, disaggregating the data by administrative regions is warranted to efficiently allocate resources where they are most needed and to reduce within-country disparities, should they exist.

A certain number of limitations should be acknowledged. First, the model does not incorporate geographical heterogeneity in HIV risk and assumed uniform spatial coverage of interventions. Spatial heterogeneity, however, could impact HIV transmission dynamics and misallocation of resources through the maintenance of residual foci of higher transmission. Second, despite the availability of recent surveys conducted among key populations, important uncertainty remained around estimates for the treatment cascade (as for the general population). For example, in a study conducted in 2011 among MSM in Abidjan, 63% of HIV-positive MSM reported having ever been tested and received their results, but only 14% reported being aware of their status [10]. This high discrepancy between the 2 estimates either entails a very high incidence among those tested, for them to have been rapidly infected after their last negative test, or selective underreporting of HIV positivity among MSM. Selective underreporting of HIV seropositivity is highly likely and has been reported in household-based surveys in Malawi and Uganda [44]. Third, some interventions were not considered—for example, pre-exposure prophylaxis was not included because it is not currently available in Côte d’Ivoire. Such interventions could further reduce HIV incidence and, adequately used, could improve the impact of the accelerated response. Finally, results for this study are likely not generalizable to countries with a very different epidemic type. Yet, Côte d’Ivoire should be broadly representative of other West African countries and an excellent case study.

Strengths of this study included that it relied on a detailed and carefully calibrated dynamic model of HIV transmission that included key populations such as FSW and MSM. Second, the model’s parameters were informed by a comprehensive review of the literature and of HIV programs [3,12]. The model enabled us to triangulate estimates from different sources, resulting in a more robust parameterization that reflects parameter uncertainty in impact estimates. Further, several behavioral surveys conducted in that country were reanalyzed to inform sexual behaviors and mixing among both age and risk groups. Finally, we investigated many different scenarios of accelerated response, providing detailed information on the factors important to reduce HIV transmission. This includes the impact of sustaining condom use, which has often been outshined by ART in the global HIV prevention discourse but remains crucial for key populations. The model provides a useful framework to investigate future lines of inquiries, including finding ways to reach those policy objectives in the most cost-efficient manner.

In conclusion, our results suggest that accelerating the response to meet the UNAIDS target could prevent a significant number of new HIV infections, especially in the long term. Avoiding delays in reaching the first 90-90-90 objective will maximize the population-level impact of the HIV response. While initiation and retention into ART programs are crucial for this modeled impact to materialize, HIV testing appears as an important bottleneck. Finally, considering MSM, FSW, and CFSW within the UNAIDS 90-90-90 response is important to efficiently reduce population-level transmission. Maintaining high condom use levels in key populations will also be central if considerable reductions in new HIV infections in the general population are to be observed.

## Supporting information

S1 Appendix This file contains the tables of parameters, additional results, and the sensitivity analyses. (DOCX)

S2 Appendix Résumé en français (French translation of the abstract). (DOCX)

## Abbreviations

AIS: AIDS Indicator Survey
ANC: antenatal care
ART: antiretroviral therapy
CFSW: client(s) of female sex workers
CrI: credible interval
DHS: Demographic Health Survey
FSW: female sex worker(s)
MSM: men who have sex with men
PF: fraction of HIV infections prevented
PLWH: people living with HIV
PMTCT: prevention of mother-to-child transmission
SC: scenario
UNAIDS: Joint United Nations Programme on HIV/AIDS
