# Supplementary material for: Population-level impact of an accelerated HIV response plan to reach the UNAIDS 90-90-90 target in Côte d’Ivoire: Insights from mathematical modeling
Source: PLoS Med. 2017 Jun 15;14(6):e1002321. doi: 10.1371/journal.pmed.1002321 (PMC5472267; doi:10.1371/journal.pmed.1002321)
Supplement: S1 Appendix — (DOCX) [file pmed.1002321.s001.docx]

**S1 Appendix**

This supplemental appendix provides complimentary information to the main paper. The full details of the model, including the model flowchart, the equations for the dynamic model, the force of infection, and the decision tree for mother-to-child transmission can be found elsewhere [1].

**Table A.** Demographic parameters and the model’s initial conditions.

| **Parameters** | **Symbol** | **Prior distributions** | **Posterior median (95% CrI)** | **References** |
| --- | --- | --- | --- | --- |
| **Initial conditions** |  |  |  |  |
| HIV prevalence in 1975 among FSW (%) | *Seed_FSW_* | *U*(0.1%, 1%) | 0.5% (0.2, 0.9%) | Assumption |
| HIV prevalence in 1975 among clients of FSW (%) | *Seed_CFSW_* | *U*(0.1%, 0.5%) | 0.3% (0.1%, 0.5%) | Assumption |
| HIV prevalence in 1975 among MSM (%) | *Seed_MSM_* | *U*(0.1%, 1%) | 0.5% (0.2%, 0.9%) | Assumption |
| Total population aged 15-59 years in 1970 | *N_0_* | 2,711,050 | NA | [2] |
| **Demography** |  |  |  |  |
| Population growth rate (year^-1^) | *ε* | 0.032 | NA | [2] |
| Immigration rate of 25-49 years old (year^-1^) | *χ* | *U*(0.017, 0.029) | 0.024 (0.019, 0.029) | [2] |
| Mortality rate (1/life expectancy at 15, 25, and 50 years) |  |  |  |  |
| 15-24 years old (year^-1^) | *μ_1:2_* | *U*(0.020, 0.021) | 0.020 (0.020, 0.021) | [2] |
| 25-49 years old (year^-1^) | *μ_3_* | *U*(0.024, 0.025) | 0.024 (0.024, 0.024) | [2] |
| 50-59 years old (year^-1^) | *μ_4_* | *U*(0.044, 0.046) | 0.045 (0.044, 0.045) | [2] |
| Fertility rate of 15-24 years old (year^-1^) | *ASFR_1:2_(t)* | Varies with time between 0.328 (1970) to 0.212 (2015) | NA | [2] |
| Fertility rate of 25-49 years old (year^-1^) | *ASFR_3_(t)* | Varies with time between 0.230 (1970) to 0.150 (2015) | NA | [2] |
| Relative fertility reduction of HIV positive women | *RR_Fert_* | *T*(0.76, 0.60, 0.89) | 0.75 (0.66, 0.83) | [3] |
| **Size of sex, age, and risk groups** |  |  |  |  |
| Proportion of males in population |  | 51.5% | NA | [2] |
| *Age class distributions* |  |  |  |  |
| 15-19 years old (%) | *PR_A_1_* | 21.0% | NA | [2] |
| 20-24 years old (%) | *PR_A_2_* | 17.5% | NA | [2] |
| 25-49 years old (%) | *PR_A_3_* | 51.5% | NA | [2] |
| 50-59 years old (%) | *PR_A_4_* | 10.0% | NA | [2] |
| Proportion of virgin females aged 15-19 years old (%) | *Vir_k_(t)* | 1994 = 27.4% | NA | [4] |
|  |  | 1999 = 35.9% | NA | [5] |
|  |  | 2005 = 34.2% | NA | [6] |
|  |  | 2012 = 35.3% | NA | [7] |
| Proportion of virgin males aged 15-19 years old (%) | *Vir_k_(t)* | 1999 = 44.3% | NA | [5] |
|  |  | 2005 = 48.9% | NA | [6] |
|  |  | 2012 = 57.3% | NA | [7] |
| Proportion of high-risk (>2 partners year^-1^) heterosexual males (%) | *PR_HRM_* | *U*(5%, 10%) | 7.6% (5.4, 9.5%) | [5-7] |
| Proportion of high-risk (>1 partner year^-1^) heterosexual females (%) | *PR_HRF_* | *U*(5%, 10%) | 7.3% (5.3, 9.6%) | [5-7] |
| Proportion of MSM among males (%) | *PR_MSM_* | *U*(0.8%, 1.6%) | 1.2% (0.9, 1.6%) | [8-10] |
| Proportion of MSM who are bisexual (%) | *PR_Bi_* | *U*(53.6%, 75.5%) | 64.9% (56.3, 73.1%) | [11,12]† |
| Ratio of FSW to MSM* | *R_FSW_* | *U*(1, 1.7) | 1.3 (1.0, 1.6) | [5,13,14] |
| Proportion of FSW that engage in AI (%) | *PR_AI_* | *U*(18.3%, 29.9%) | 24.5% (19.6, 28.8%) | Based on [15] |
| Turnover rate from FSW to non-FSW (year^-1^) | *tur* | *U*(1/15, 1/5) | 0.11 (0.07, 0.18) | Assumption |

AI=anal intercourse; FSW=female sex worker; MSM=men who have sex with men; *T*(m,a,b)=Triangular distribution (m=mode, a=minimum, b=maximum); *U*(a,b)=Uniform distribution (a=minimum, b=maximum).

*The proportion of clients of FSW was indirectly estimated using the multiplier method, balancing the partner change rate reported by FSW and by clients of FSW. The proportion of clients of FSW was constrained to be less than 20%.

†Parameter informed by the analysis of primary data of respondent-driven sampling surveys conducted in five cities of Côte d’Ivoire (unpublished).

**Table B.** Behavioral parameters.

| **Parameters** | **Symbols** | **Prior distributions or data** | **Posterior median (95% CrI)** | **References** |
| --- | --- | --- | --- | --- |
| **Partner change rate of sexually active** |  |  |  |  |
| Low-risk females (year^-1^) | *c_1_* | *U*(0.8, 0.9) | 0.85 (0.81, 0.90) | [5-7] |
| High-risk females (year^-1^) | *c_2_* | *U*(2.4, 9.4) | 6.0 (2.9, 8.7) | [5-7] |
| FSW (year^-1^) | *c_3:4_* | *U*(216, 360) | 284 (225, 348) | [16-19]* |
| Low-risk males (year^-1^) | *c_5_* | *U*(1.0, 1.2) | 1.1 (1.0, 1.2) | [5-7] |
| High-risk males (year^-1^) | *c_6_* | *U*(4.7, 6.8) | 5.8 (4.8, 6.7) | [5-7] |
| Clients of FSW (with FSW) (year^-1^) | *c_7_* | *U*(23, 37) | 31 (24, 36) | [20] |
| Clients of FSW (with women in the general population) (year^-1^) | *c_7b_* | *U*(1, 6.8) | 5.4 (3.6, 6.5) | [5-7] |
| Bisexual MSM (year^-1^) | *c_8_* | *U*(1, 10) | 2.2 (1.1, 3.9) | Assumption |
| Exclusive MSM (year^-1^) | *c_9_* | *U*(1, 10) | 2.5 (1.1, 5.1) | [12] |
| **Number of sex acts** |  |  |  |  |
| Low-risk partners (partnership^-1^ year^-1^) | *α_kijl_* | *U*(40, 48) | 45 (41, 48) | [7] |
| High-risk partners (partnership^-1^ year^-1^) | *α_kijl_* | *U*(33, 66) | 51 (38, 63) | [7] |
| MSM partners (partnership^-1^ year^-1^) | *α_kijl_* | *U*(33, 66) | 44 (34, 58) | Assumption |
| Clients-FSW partners (partnership^-1^ year^-1^) | *α_kijl_* | *U*(1, 4) | 3 (2, 3) | [7] |
| **Sexual mixing parameters** |  |  |  |  |
| Sexual balance parameter as per Garnett et al. 1994 [21]† | *η* | *U*(0, 1) | 0.12 (0.01, 0.28) | Assumption |
| Fraction of partnerships that are with females for bisexual males (%) | *Bi_Pref_* | *U*(32%, 44%) | 38% (33, 43%) | Primary data‡ |
| Tuning parameter between assortative and proportional mixing by age among MSM | *MSM_AgeMix_* | *U*(0, 1) | 0.61 (0.20, 0.96) | Assumption |
| **Other parameters** |  |  |  |  |
| Proportion of sex acts that are anal for FSW that engage in AI (%) | *Pr_ActsAI_* | *U*(14.8%, 27.6%) | 0.210 (0.153, 0.263) | Based on [15,17] |

AI=anal intercourse; CFSW=clients of female sex workers; DHS=Demographic and Health Survey; FSW=female sex worker; MSM=men who have sex with men; *U*(a,b)=Uniform distribution (a=min, b=max).

*Based on the assumption that FSW engage in sex work 4 days per week, 50 weeks per year.

†CFSW were assumed to drive demand and FSW were de facto assumed to adjust their partner change rate in consequence. For MSM, we assumed that the balance parameter is equal to 0.5.

‡ Parameter informed by the analysis of primary data of respondent-driven sampling surveys conducted in five cities of Côte d’Ivoire (unpublished).

**Table C.** Biological and treatment parameters.

| **Parameters** | **Symbols** | **Prior distributions or data** | **Posterior median (95% CrI)** | **References** |
| --- | --- | --- | --- | --- |
| **HIV transmission probability** |  |  |  |  |
| Female-to-male transmission probability (per VI act^-1^) | *β_fm_* | *U*(0.00013, 0.00141) | 0.00089 (0.00056, 0.00128) | [22] |
| Male-to-female transmission probability (per VI act^-1^) | *β_mf_* | *U*(0.00060, 0.00109) | 0.00089 (0.00063, 0.00106) | [22] |
| **Changes in HIV transmission probability** |  |  |  |  |
| RR of HIV acquisition for receptive AI as compared to receptive VI (act^-1^) | *RR_RAI_* | *T*(10, 2, 20) | 6.3 (3.0, 11.1) | [23] |
| RR of HIV acquisition for insertive AI as compared to insertive VI (act^-1^) | *RR_IAI_* | 2 | NA | [23] |
| RR of HIV acquisition for women 15-24 years old as compared to those aged ≥25 years (act^-1^) | *RR_YoungFem_* | *U*(1.25, 2.5) | 2.0 (1.5, 2.4) | [24,25] |
| RR of HIV transmission during acute infection (act^-1^) | *RR_Acute_* | *T*(9.2, 4.5, 18.8) | 8.6 (5.6, 12.6) | [22] |
| RR of HIV transmission for individuals on ART (detectable viral load) (act^-1^) | *RR_VLD_* | *T*(0.50, 0.30, 0.80) | 0.54 (0.40, 0.72) | Assumption |
| RR of HIV transmission for individuals on ART (virally suppressed; <50 copies/μL) (act^-1^) | *RR_ART_* | *T*(0.04, 0.01, 0.27) | 0.12 (0.03, 0.22) | [26] |
| RR of HIV transmission when sex act is protected by a condom (act^-1^) | *ϛ* | *U*(0.75, 0.942) | 0.83 (0.77, 0.91) | [27] |
| **Natural history progression** |  |  |  |  |
| Time duration of acute infection (years) | *1/γ_1_* | *U*(0.16, 0.50) | 0.33 (0.18, 0.47) | [28] |
| Time duration from seroconversion to 350 CD4 cells/μL (years) | *1/γ_1_+1/γ_2_+1/γ_3_* | *U*(2.2, 4.6) | 3.9 (2.8, 4.5) | [29] |
| Time duration from 350 CD4 cells/μL to 200 CD4 cells/μL (years) | *1/γ_4_* | *U*(3.9, 5.0) | 4.5 (4.0, 4.9) | [29] |
| Time duration from 200 CD4 cells/μL to death (years) | *1/γ_5_* | *U*(1.9, 3.9) | 2.9 (2.1, 3.8) | [29] |
| Time duration from unsuppressed to suppressed viral load (<50 copies/μL) (years) (probability converted to duration using *1/–ln(1-0.871)/t*) | *ϑ* | 0.49 | NA | [30] |
| **ART** |  |  |  |  |
| Survival extension cofactor for individuals on ART (RR)* | *RR_ω_* | *U*(2, 3) | 2.5 (2.1, 2.9) | [31,32] |
| Rate of therapeutic failures (years^-1^) | *φ* | *U*(0.049, 0.09) | 0.070 (0.054, 0.088) | Based on [33,34] |
| Rate of ART discontinuation (years^-1^) (probability converted to rate using *–ln(1-P)/t*) | *l* | *U*(0.15, 0.27) | 0.21 (0.16, 0.27) | [35-40] |
| Relative risk of ART discontinuation for FSW | *RR_ARTD-FSW_* | *U*(1.25, 1.75) | 1.50 (1.28, 1.73) | [41] |
| Relative risk of ART discontinuation for MSM | *RR_ARTD-MSM_* | *U*(1.25, 1.75) | 1.49 (1.30, 1.71) | Assumed equal to that of FSW |

AI=anal intercourse; ART=antiretroviral therapy; FSW=female sex worker; MSM=men who have sex with men; RR=relative risk; *T*(m,a,b)=Triangular distribution (m=mode, a=minimum, b=maximum); *U*(a,b)=Uniform distribution (a=minimum, b=maximum); VI=vaginal intercourse.

*HIV-related mortality (*ω_s_*) for those on treatment is calculated as followed: *ω_s_=(Σγ_s_)/RR_ω._*

**Table D.** Mother-to-child HIV transmission probabilities as a function of the availability (or lack thereof) of different interventions, CD4 cell counts, and breastfeeding status.

| **Parameters** | **Symbols** | **Prior distributions or data** | **Posterior median (95% CrI)** | **References** |
| --- | --- | --- | --- | --- |
| **No prophylaxis (prior to 2000)** |  |  |  |  |
| Peripartum transmission probability (CD4<200 cells/μL) | *β_<200_^0^* | *U*(0.22, 0.54) | 0.39 (0.24, 0.53) | [42] |
| Peripartum transmission probability (CD4 200-349 cells/μL) | *β_<350_^0^* | *U*(0.131, 0.326) | 0.23 (0.14, 0.31) | [42] |
| Peripartum transmission probability (CD4≥350 cells/μL) | *β_≥350_^0^* | *U*(0.097, 0.202) | 0.15 (0.11, 0.20) | [42] |
| Monthly postnatal transmission probability for any breastfeeding (CD4<350 cells/μL) | *β_BF<350_^0^* | 0.0157 | NA | [42] |
| Monthly postnatal transmission probability for any breastfeeding (CD4≥350 cells/μL) | *β_BF≥350_^0^* | 0.0051 | NA | [42] |
| **Single-dose nevirapine (from 2000 to 2005)** |  |  |  |  |
| Peripartum transmission probability | *β_Px_^1^* | *U*(0.094, 0.121) | 0.11 (0.10, 0.12) | [42] |
| Monthly postnatal transmission probability for any breastfeeding (CD4<350 cells/μL) | *β_BF<350_^1^* | 0.0157 | NA | [42] |
| Monthly postnatal transmission probability for any breastfeeding (CD4≥350 cells/μL) | *β_BF≥350_^1^* | 0.0051 | NA | [42] |
| **Dual prophylaxis (from 2005 to 2010)** |  |  |  |  |
| Peripartum transmission probability | *β_Px_^2^* | *U*(0.023, 0.053) | 0.039 (0.025, 0.050) | [42] |
| Monthly postnatal transmission probability for any breastfeeding (CD4<350 cells/μL) | *β_BF<350_^2^* | 0.0157 | NA | [42] |
| Monthly postnatal transmission probability for any breastfeeding (CD4≥350 cells/μL) | *β_BF≥350_^2^* | 0.0051 | NA | [42] |
| **WHO’s option A or B (from July 2010 onward)** |  |  |  |  |
| Peripartum transmission probability | *β_Tx_^3^* | *U*(0.009, 0.029) | 0.019 (0.011, 0.027) | [42] |
| Monthly postnatal transmission probability for any breastfeeding |  | 0.002 | NA | [42] |
| **Women already on ART** |  |  |  |  |
| Peripartum transmission probability | *β_ARV_* | 0.005 | NA | [42] |
| Monthly postnatal transmission probability for any breastfeeding |  | 0.0016 | NA | [42] |
| **Breastfeeding** |  |  |  |  |
| Proportion of infants not being breastfeed (%) | *(1-A)* | *U*(1.4%, 3.6%) | 2.4 (1.5, 3.4%) | [5-7] |
| Average duration of any breastfeeding (months) | *D* | *U*(18.8, 20.5) | 19.7 (18.9, 20.3) | [5-7] |

ART=antiretroviral therapy; *U*(a,b)=Uniform distribution (a=minimum, b=maximum); WHO=World Health Organization.

**Table E**. Parameters used to estimate historical trends in the proportion of sex acts protected by a condom.

| **Parameters** | **Symbols** | **Prior distributions/ ranges or data** | **Posterior median (95% CrI)** | **References** |
| --- | --- | --- | --- | --- |
| **General population (excluding CFSW, FSW, MSM)** | | | | |
| 15-24 years old (%) | *Condom_1:2_(t)* | 1981 = (0%, 5%) | Figure I | [43,44] |
| (Proportion of protected sex acts) | | 1994 = (9.9%, 47.2%) |  | [4]* |
|  |  | 1999 = (11.5%, 55.7%) |  | [5]* |
|  |  | 2005 = (20.9%, 53.5%) |  | [6]* |
|  |  | 2012 = (20.7%, 59.5%) |  | [7]* |
| 25-49 years old (%) | *Condom_3_(t)* | 1981 = (0%, 2.5%) | Figure I | [43,44] |
|  |  | 1994 = (2.5%, 21.7%) |  | [4]* |
|  |  | 1999 = (3.1%, 21.8%) |  | [5]* |
|  |  | 2005 = (4.7%, 23.8%) |  | [6]* |
|  |  | 2012 = (7.3%, 24.2%) |  | [7]* |
| 50-59 years old (%) | *Condom_4_(t)* | 1981 = (0%, 2.0%) |  | [43,44] |
|  |  | 1994 = (0.2%, 9.7%) |  | [4]***†** |
|  |  | 1999 = (0.6%, 9.7%) |  | [5]***†** |
|  |  | 2005 = (0.6%, 10.0%) |  | [6]***†** |
|  |  | 2012 = (1.8%, 11.5%) |  | [7]***†** |
| **Female sex workers with their clients (VI)** | | | | |
| All age groups (%) | *CondomSW(t)* | 1981 = (0%, 5.0%) | Figure I | Based on [43,44] |
| (Proportion of protected sex acts) | | 1991 = (57%, 68%) |  | [16] |
|  |  | 1993 = (74%, 81%) |  | [16] |
|  |  | 1995 = (73%, 88%) |  | [16] |
|  |  | 1997 = (88%, 93%) |  | [16] |
|  |  | 1998 = (88%, 98%) |  | [19] |
|  |  | 2002 = (91%, 99%) |  | [19] |
|  |  | 2007 = (90%, 99%) |  | [19] |
|  |  | 2011 = (90%, 95%) |  | [19] |
|  |  | 2014 = (85%, 93%) |  | [15,17] |
| **Relative reduction in condom use for FSW AI versus VI** | | | | |
| All age groups (RR) | *RR_CondAI_* | *T*(0.75, 0.61, 0.92) | 0.75 (0.66, 0.85) | [15,17] |
| **Men who have sex with men** | |  |  |  |
| All age groups (%) | *CondomMSM(t)* | 1981 = 0% | Figure I | Assumption |
| (Proportion of protected sex acts) | | 2004 = (35%, 50%) |  | Based on [45] |
|  |  | 2012 = (57%, 69%) |  | [11] |
|  |  | 2015 = (63%, 81%) |  | § |
| **Year of condom increase in the 1980s** | |  |  |  |
| General population (k=1,2,5,6) | *DateCond_k_* | *U*(1981,1990) | 1986 (1982, 1989) | Assumption |
| Sex work (k=3,4,7) | *DateCond_k_* | *U*(1981,1990) | 1987 (1982, 1990) | Assumption |
| MSM (k=8,9) | *DateCond_k_* | *U*(1981,1990) | 1986 (1981, 1989) | Assumption |
| **Scaling factor for the proportion of sex acts protected by condoms‡** | | | | |
| 15-24 years old (general population) | *CondPtl_1:2_* | *U*(0, 1) | 0.52 (0.07, 0.93) | Assumption |
| 25-49 years old (general population) | *CondPtl_3_* | *U*(0, 1) | 0.47 (0.04, 0.84) | Assumption |
| 50-59 years old (general population) | *CondPtl_4_* | *U*(0, 1) | 0.56 (0.11, 0.92) | Assumption |
| FSW (all age groups) | *CondSWPtl* | *U*(0, 1) | 0.44 (0.06, 0.85) | Assumption |
| MSM (all age groups) | *CondMSMPtl* | *U*(0, 1) | 0.55 (0.08, 0.92) | Assumption |

AI=anal intercourse; CFSW=client of female sex worker; FSW=female sex worker; MSM=men who have sex with men; RR=relative risk; *T*(m,a,b)=Triangular distribution (m=mode, a=minimum, b=maximum); *U*(a,b)=Uniform distribution (a=minimum, b=maximum); VI=vaginal intercourse.

*The minimum of the range corresponds to the lower confidence bound of the estimate reported by females and the maximum to the upper confidence bound of the estimate reported by males.

†Only the 2005 AIDS Indicator Survey collected information on men aged 50-59 years old. When unavailable, estimates for this age class were approximated by extrapolating those of the 45-49 years old age group.

**‡**The estimates of the proportion of sex acts protected by a condom are sampled for each year using the same percentile of the distribution to obtain temporally consistent estimates.

§Parameter informed by the analysis of primary data of respondent-driven sampling surveys conducted in five cities of Côte d’Ivoire (unpublished).

**Table F**. Parameters used to estimate historical trends in HIV testing.

| **Parameters** | **Symbols** | **Prior distributions/ranges or data** | **Posterior median (95% CrI)** | **References** |
| --- | --- | --- | --- | --- |
| **HIV testing probability (last 12 months) for asymptomatic females (excluding those tested during ANC)*** | | | | |
| Among 15-24 years old (year^-1^) | *τ_k1:2_^0^(t)* | 2000 = 0% | 0% | Based on [46] |
|  |  | 2005 = (1.1%, 2.4%) | 1.6% (1.1, 2.2%) | [6] |
|  |  | 2012 = (6.3%, 8.6%) | 7.1% (6.4, 8.2%) | [7] |
| Among 25-49 years old (year^-1^) | *τ_k3_^0^(t)* | 2000 = 0% | 0% | Based on [46] |
|  |  | 2005 = (1.8%, 4.5%) | 2.9% (1.9, 4.0%) | [6] |
|  |  | 2012 = (6.0%, 8.0) | 6.8% (6.1-7.6%) | [7] |
| Among 50-59 years old (year^-1^) | *τ_k4_^0^(t)* | 2000 = 0% | 0% | Assumption |
|  |  | 2005 = (0.1%, 2.7%) | 1.1% (0.2, 2.2%) | [6]† |
|  |  | 2012 = (4.1%, 8.0%) | 5.7% (4.2, 7.3%) | [7]† |
| **HIV testing probability (last 12 months) for asymptomatic males*** | | | | |
| Among 15-24 years old (year^-1^) | *τ_k1:2_^0^(t)* | 2000 = 0% | 0% | Based on [46] |
|  |  | 2005 = (1.3%, 3.6%) | 2.2% (1.4, 3.2%) | [6] |
|  |  | 2012 = (5.8%, 9.7%) | 7.4% (5.9, 9.0%) | [7] |
| Among 25-49 years old (year^-1^) | *τ_k3_^0^(t)* | 2000 = 0% | 0% | Based on [46] |
|  |  | 2005 = (2.9%, 5.4%) | 3.9% (3.0, 5.0%) | [6] |
|  |  | 2012 = (8.9%, 13.1%) | 10.6% (9.1, 12.4%) | [7] |
| Among 50-59 years old (year^-1^) | *τ_k4_^0^(t)* | 2000 = 0% | 0% | Assumption |
|  |  | 2005 = (0.8%, 2.9%) | 1.7% (0.9, 2.7%) | [6]† |
|  |  | 2012 = (3.1%, 8.1%) | 5.1% (3.3, 7.2%) | [7]† |
| **Proportion of pregnant women accessing ANC and being tested for HIV‡** | | | | |
| Among 15-24 years old (year^-1^) | *ANCτ_1:2_(t)* | 1999 = 0 | 0% | Based on [46] |
|  |  | 2005 = (4.6%, 10.9%) | 7.1% (4.8, 9.8%) | [6] |
|  |  | 2012 = (32.1%, 41.1%) | 35.7% (32.4, 39.5%) | [7] |
| Among 25-49 years old (year^-1^) | *ANCτ_3_(t)* | 1999 = 0 | 0% | Based on [46] |
|  |  | 2005 = (10.9%, 18.9%) | 14.1% (11.2, 17.5%) | [6] |
|  |  | 2012 = (36.6%, 44.9%) | 39.9% (36.9, 43.4%) | [7] |
| **Relative increase in testing rate for different groups** | | | | |
| Symptomatic stage (<200 CD4 cells/μL) (RR) | *Symp_RR_Test_* | *U*(2, 8) | 4.8 (2.3, 7.8) | Assumption |
| Female sex worker (RR) | *FSW_RR_Test_* | *U*(1, 4) | 2.5 (1.2, 3.7) | Based on [7,19] |
| Men who have sex with men (RR) | *MSM_RR_Test_* | *U*(1, 4) | 2.3 (1.2, 3.7) | Based on [7,11] |
| **Scaling factor for HIV testing§** | | | | |
| All age and risk groups (excluding ANC) | *TestPtl* | *U*(0, 1) | 0.40 (0.04, 0.82) | Assumption |
| Pregnant women tested during ANC | *ANCPtl* | *U*(0, 1) | 0.50 (0.08, 0.95) | Assumption |

ANC=antenatal care; FSW=female sex worker; MSM=men who have sex with men; RR=relative risk; *T*(m,a,b)=Triangular distribution (m=mode, a=minimum, b=maximum); *U*(a,b)=Uniform distribution (a=minimum, b=maximum); VI=vaginal intercourse.

*Probabilities of being tested during last 12 months was converted to rate (year^-1^) using Rate = -ln(1-Probability)

†Only the 2005 AIDS Indicator Survey collected information on men aged 50-59 years old. When unavailable, estimates for this age class were approximated by extrapolating those of the 45-49 years old age group.

**‡**Women aged 50-59 years old are assumed to have a fertility rate of zero.

**§**The estimates for HIV testing rate and proportion of pregnant women tested as part of their ANC are sampled for each year using the same percentile of the distribution to obtain temporally consistent estimates.

**Table G**. Parameters used to estimate historical trends in the availability of interventions for the prevention of mother-to-child transmission.

| **Parameters** | **Symbols** | **Prior distributions/ranges or data** | **Posterior median (95% CrI)** | **References** |
| --- | --- | --- | --- | --- |
| **Pregnant women tested positive for HIV during ANC receiving ART prophylaxis (%)*** | | | | |
|  | *Κ(t)* | 1999 = 0% | 0% | Assumption |
|  |  | 2002 = (40.5%, 53.4%) | 47.4% (41.4, 52.6%) | [47] |
|  |  | 2007 = (51.7%, 65.4%) | 59.0% (52.6, 64.6%) | [35] |
|  |  | 2008 = (37.3%, 62.4%) | 50.7% (39.0, 60.9%) | [35] |
|  |  | 2009 = (44.2%, 61.5%) | 53.5% (45.4, 60.4%) | [36] |
|  |  | 2010 = (47.5%, 59.5%) | 53.9% (48.3, 58.8%) | [37] |
|  |  | 2011 = (53.0%, 60.5%) | 57.0% (53.5, 60.0%) | [38] |
|  |  | 2012 = (43.4%, 55.8%) | 50.0% (44.2, 55.0%) | [39] |
|  |  | 2013 = (41.1%, 55.8%) | 49.0% (42.1, 54.9%) | [40] |
|  |  | 2014 = (49.6%, 66.5%) | 58.6% (50.7, 65.5%) | [48] |
| **Pregnant women tested positive for HIV during ANC receiving ART treatment (%)**† | | | | |
|  | *Φ(t)* | 1999 = 0% | 0% | Assumption |
|  |  | 2008 = (9.6%, 11.9%) | 10.7% (9.7, 11.7%) | [35] |
|  |  | 2009 = (10.4%, 10.7%) | 10.5% (10.4, 10.7%) | [36] |
|  |  | 2010 = (10.7, 13.2%) | 11.8% (10.8, 13.0%) | [37] |
|  |  | 2011 = (11.9, 14.7%) | 13.2% (12.0, 14.4%) | [38] |
|  |  | 2012 = (11.9, 14.7%) | 13.2% (12.0, 14.4%) | [39] |
|  |  | 2013 = (18.8%, 23.2%) | 21.8% (19.0, 22.8%) | [40] |
|  |  | 2014 = (22.5%, 27.8%) | 24.9% (22.7 27.3%) | [48] |
| **Scaling factor for PMTCT coverage‡** | | | | |
| ART prophylaxis | *ARTPxPtl* | *U*(0, 1) | 0.54 (0.07, 0.94) | Assumption |
| ART treatment | *ARTTxPtl* | *U*(0, 1) | 0.46 (0.04, 0.91) | Assumption |

ANC=antenatal care; ART=antiretroviral therapy; PMTCT=prevention of mother-to-child transmission.

*The lower bound of the confidence interval corresponds to the proportion of infants receiving prophylaxis and the upper bound to the proportion of mothers receiving prophylaxis.

†The bound of the prior distribution were constructed by varying point estimates by ± 10%.

**‡**The estimates for proportion of pregnant women testing positive for HIV receiving prevention of mother-to-child transmission interventions are sampled for each year using the same percentile of the distribution to temporally consistent estimates.

**Table H**. Parameters used to estimate historical trends in antiretroviral therapy coverage.

| **Parameters** | **Symbols** | **Prior distributions** | **Posterior median (95% CrI)** | **References** |
| --- | --- | --- | --- | --- |
| Recruitment rate into ART when symptomatic (<200 CD4 cells/μL) (years^-1^) | *ρ_5_* | *U*(0.5, 4) | 1.4 (0.6, 2.5) | Assumption |
| Slope cofactor to define linear relation between CD4 stages and recruitment into ART | *ι* | *U*(0, 1) | 0.58 (0.10, 0.92) | Assumption |

ART=antiretroviral treatment; *U*(a,b)=Uniform distribution (a=minimum, b=maximum).

**Table I.** Cross-validation of the Côte d’Ivoire mathematical model’s predictions as compared to UNAIDS estimates for 2015.

| **Epidemiological Variables** | **Côte d’Ivoire Model** | **UNAIDS (Spectrum)*** |
| --- | --- | --- |
| HIV Prevalence (15-49 years) | 2.9% (2.3-3.6%) | 3.2% (2.7-3.6%) |
| HIV Incidence (15-49 years) | 0.0023 (0.0018-0.0032) | 0.0019 (0.0013-0.0025) |
| New Pediatric HIV Infections† | 3,800 (2,700-5,400) | 3,600 (2,600-4,600) |
| HIV-related deaths (≥15 years)§ | 24,000 (18,000-30,000) | 25,000 (20,000-30,000) |

*Data accessed 2017-02-20 (http://aidsinfo.unaids.org/#).

†UNAIDS data included all new infections among children aged 0-14 years of age.

§The Côte d’Ivoire model includes the number of HIV deaths among the 15-59 years old.


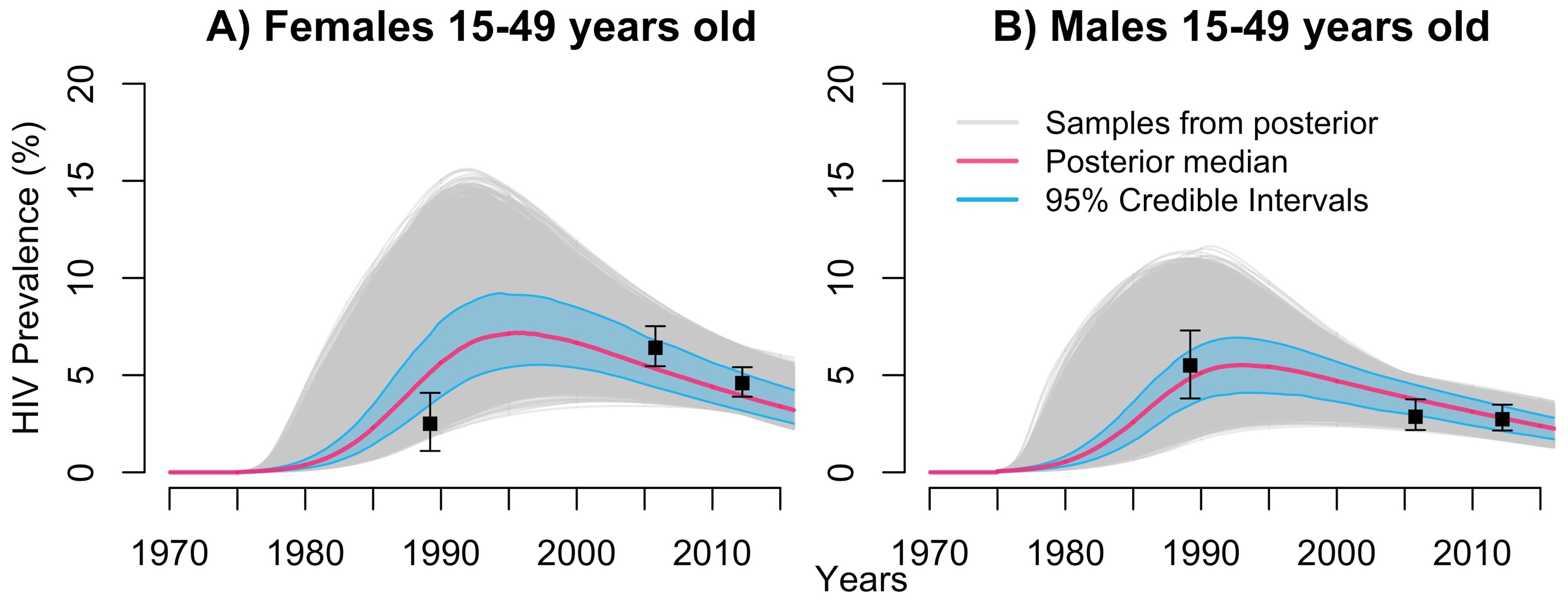


**Figure A. Observed HIV prevalence [6,7,49], to which the model was calibrated to (mean and 95% confidence intervals), and predicted HIV prevalence among A) women and B) men in Côte d’Ivoire among.** FSW are not assumed to be sampled in the household-based HIV prevalence surveys and were excluded in calculating model-based overall prevalence estimates. The 1989 survey grouped together individuals aged 25 to 54 years of age. Incremental Mixture Importance Sampling (IMIS) resulted in 41,146 unique sets of parameters labelled samples from posterior (grey curves). One thousand of these curves were resampled with probability proportional to their importance weights to derive the median (red) and 95% credible intervals (shaded blue area). Additional model fits can be found in Maheu-Giroux et al. [1].


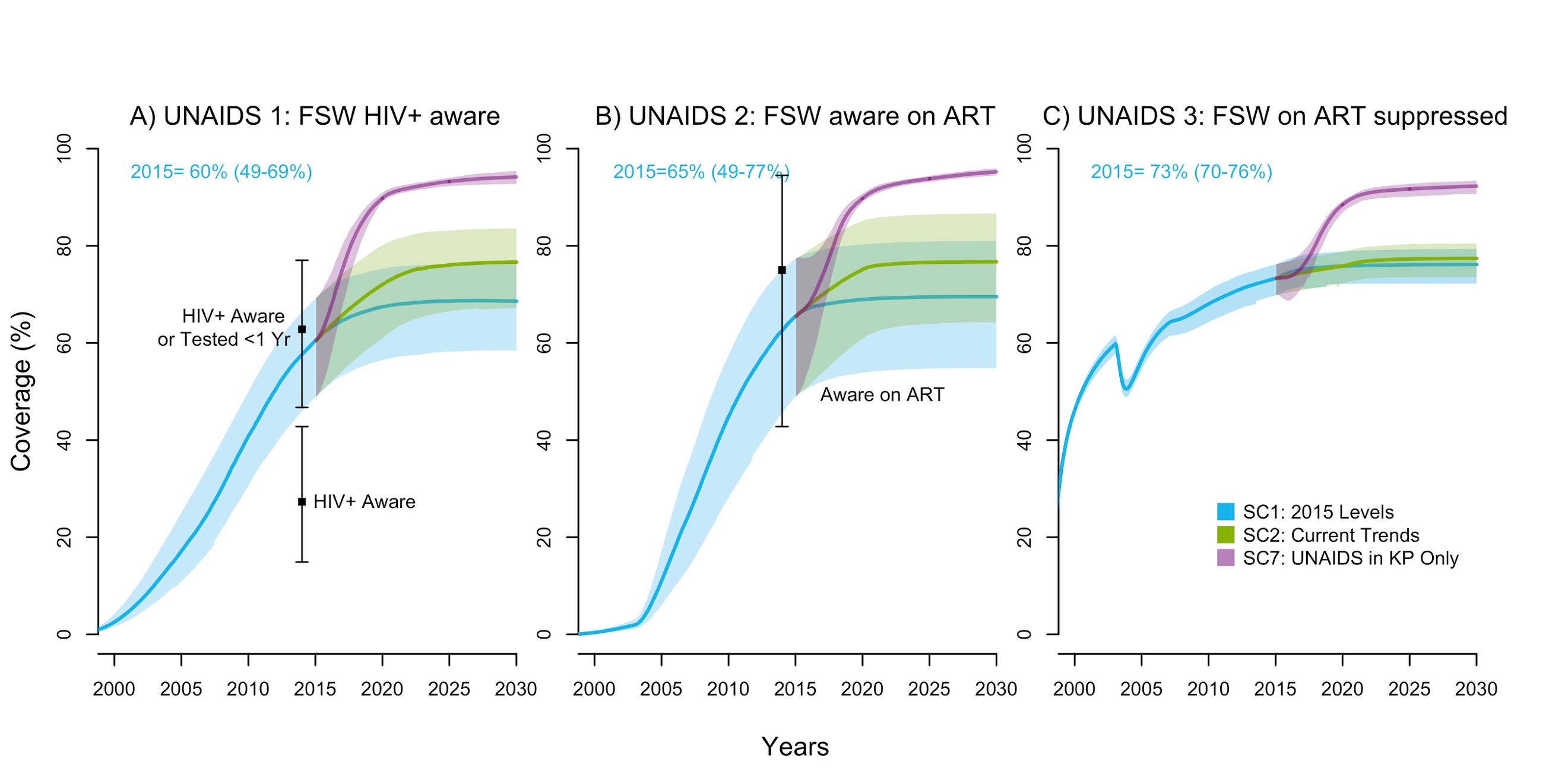


**Figure B. Coverage of the three UNAIDS indicators among female sex workers (FSW) under three intervention scenarios (median and 95% credible intervals).** UNAIDS indicators from left to right are: A) proportion of HIV positives individuals aware of their status (data from [15], used for cross-validation), B) proportion of those aware that are receiving antiretroviral therapy (ART) (data from [15], used for cross-validation), and C) proportion of those on ART virally suppressed. The scenarios are as defined in table 1. SC1) Baseline: testing rate, antiretroviral (ART) recruitment rate, and ART failure rate stable at their 2015 values; SC2) observed increase in those three rates from 2010-2015 projected through 2020; SC7) UNAIDS in key populations: 90-90-90 objective reached in 2020 and 95-95-95 in 2030 among FSW and MSM populations only.

**
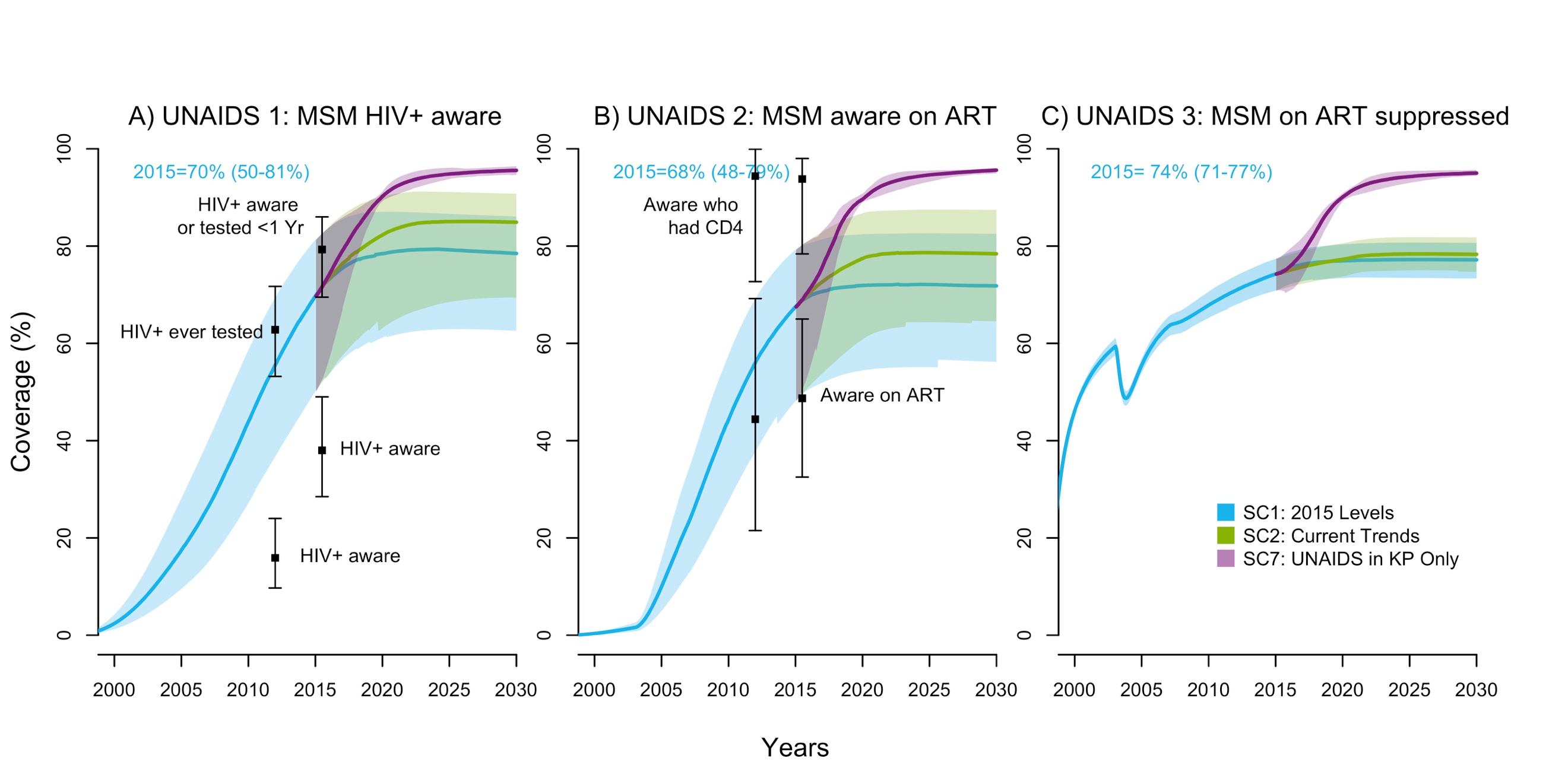
**

**Figure C. Coverage of the three UNAIDS indicators among men who have sex with men (MSM) under three intervention scenarios (median and 95% credible intervals).** UNAIDS indicators from left to right are: A) proportion of HIV positives individuals aware of their status (2012 data from [11] and 2015 unpublished data from S Baral, used for cross-validation), B) proportion of those aware that are receiving antiretroviral therapy (ART) (2012 data from [11] and 2015 unpublished data from S Baral, used for cross-validation), and C) proportion of those on ART virally suppressed. The scenarios are as defined in table 1. SC1) Baseline: testing rate, antiretroviral (ART) recruitment rate, and ART failure rate stable at their 2015 values; SC2) observed increase in those three rates from 2010-2015 projected through 2020; SC7) UNAIDS in key populations: 90-90-90 objective reached in 2020 and 95-95-95 in 2030 among FSW and MSM populations only.


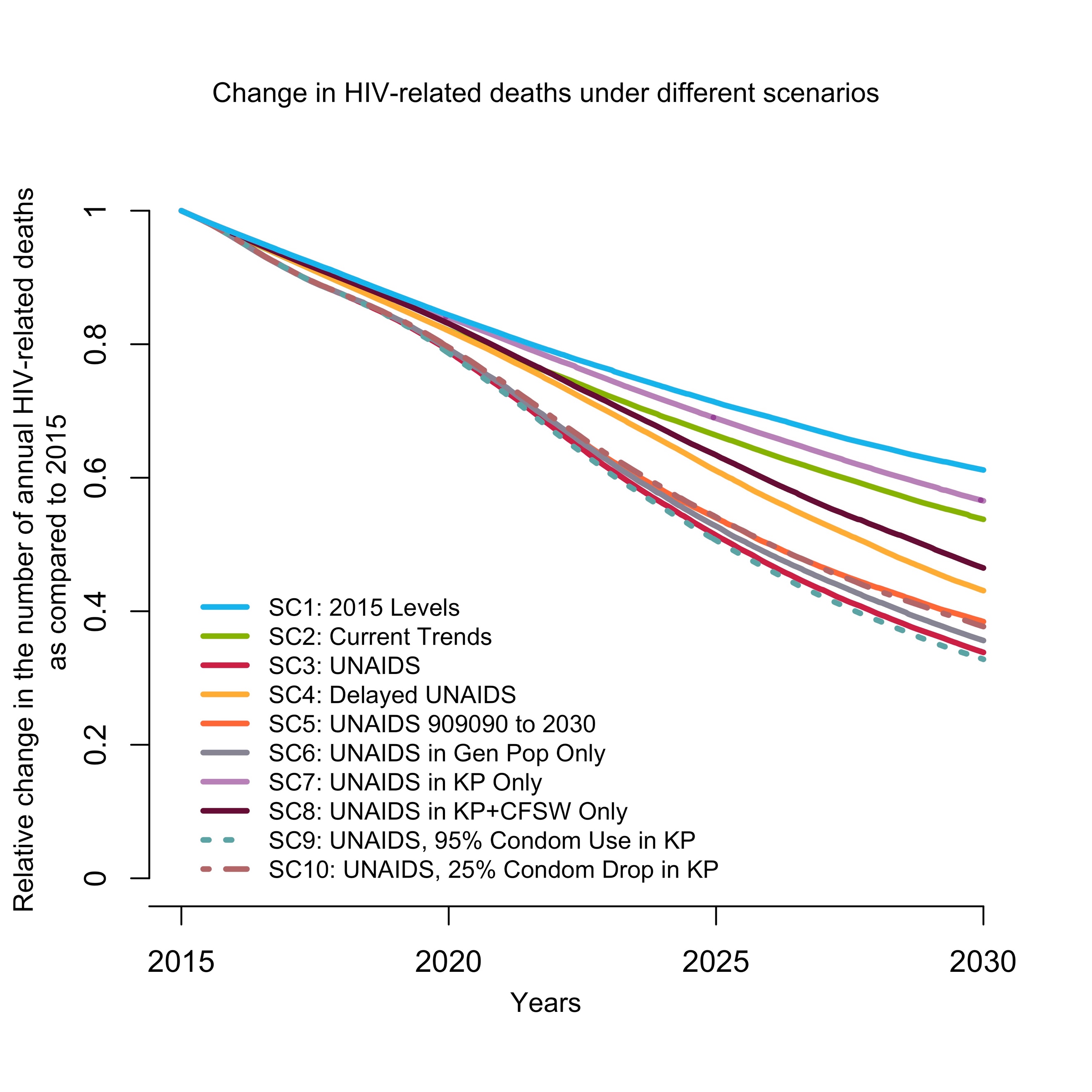


**Figure D. Predicted relative median change in annual HIV-related deaths among 15-59 years old in Côte d’Ivoire from 2015 to 2030 under different intervention coverage scenarios.** The scenarios are detailed in Table 1. SC1) Baseline: testing rate, antiretroviral (ART) recruitment rate, and ART failure rate stable at their 2015 values; SC2) Current trends: observed increase in those three rates from 2010-2015 projected through 2020; SC3) UNAIDS: 90-90-90 objective reached in 2020 and 95-95-95 in 2030; SC4) Delayed UNAIDS: 90-90-90 objective reached in 2025 and maintained to 2030; SC5) UNAIDS 909090 to 2030: 90-90-90 objective reached in 2020 and maintained to 2030, SC6) UNAIDS in general population: 90-90-90 objective reached in 2020 and 95-95-95 in 2025 among general population only; SC7) UNAIDS in key populations: 90-90-90 objective reached in 2020 and 95-95-95 in 2030 among FSW and MSM populations only; SC8) UNAIDS in key populations and CFSW: 90-90-90 objective reached in 2020 and 95-95-95 in 2030 among MSM, FSW, and CFSW only; SC9) UNAIDS plus condom in key populations: 90-90-90 objective reached in 2020 and 95-95-95 in 2030 and rise to 95% by 2020 of sexual acts protected by a condom among FSW and MSM; SC10) UNAIDS with condom drop in key populations: 90-90-90 objective reached in 2020 and 95-95-95 in 2030 and decline by 25 percentage point of sexual acts protected by a condom among FSW and MSM. (95% Credible Intervals are not presented to ease visual interpretation.)


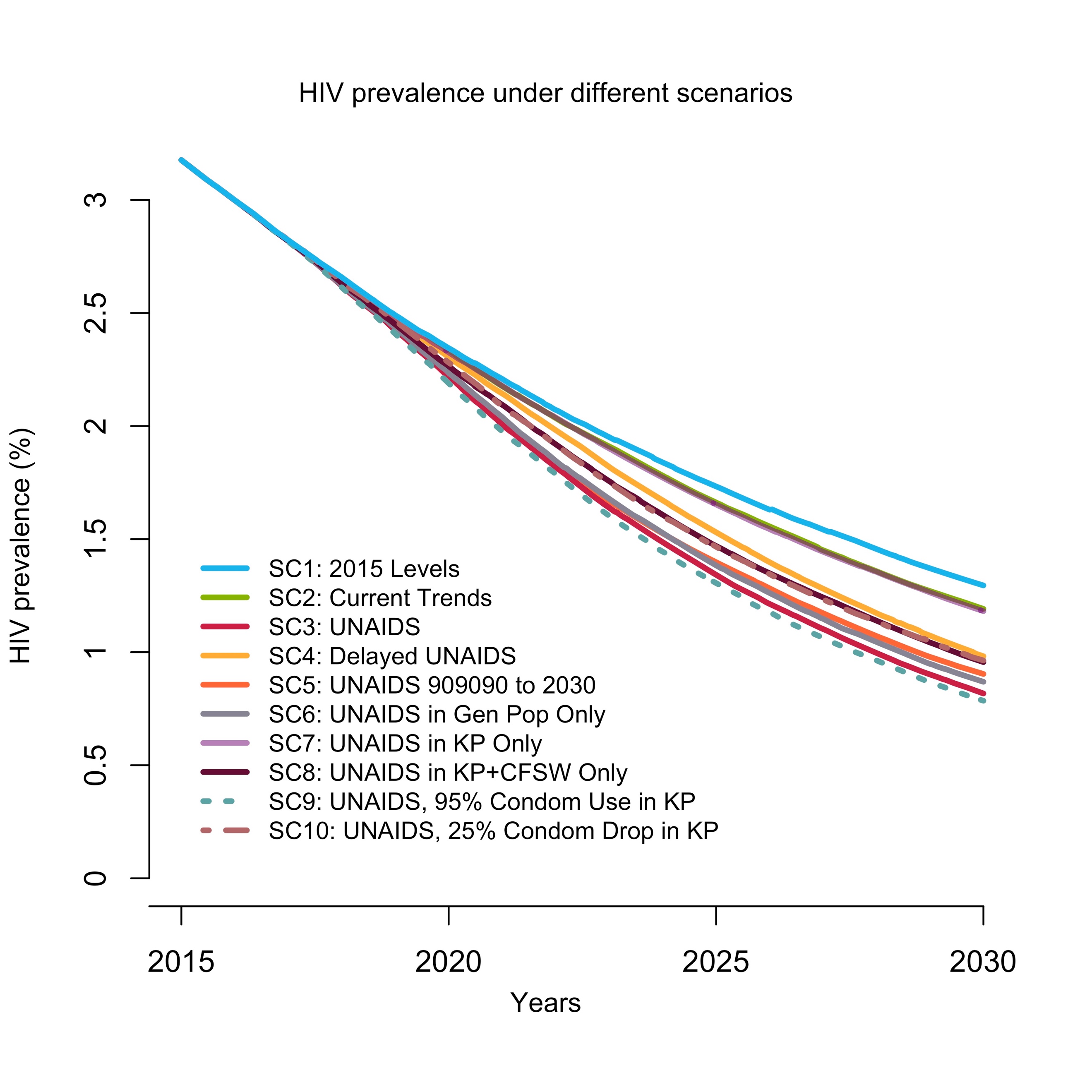


**Figure E. Predicted median HIV prevalence among 15-59 years old in Côte d’Ivoire from 2015 to 2030 under different intervention coverage scenarios.** The scenarios are detailed in Table 1. SC1) Baseline: testing rate, antiretroviral (ART) recruitment rate, and ART failure rate stable at their 2015 values; SC2) Current trends: observed increase in those three rates from 2010-2015 projected through 2020; SC3) UNAIDS: 90-90-90 objective reached in 2020 and 95-95-95 in 2030; SC4) Delayed UNAIDS: 90-90-90 objective reached in 2025 and maintained to 2030; SC5) UNAIDS 909090 to 2030: 90-90-90 objective reached in 2020 and maintained to 2030, SC6) UNAIDS in general population: 90-90-90 objective reached in 2020 and 95-95-95 in 2025 among general population only; SC7) UNAIDS in key populations: 90-90-90 objective reached in 2020 and 95-95-95 in 2030 among FSW and MSM populations only; SC8) UNAIDS in key populations and CFSW: 90-90-90 objective reached in 2020 and 95-95-95 in 2030 among MSM, FSW, and CFSW only; SC9) UNAIDS plus condom in key populations: 90-90-90 objective reached in 2020 and 95-95-95 in 2030 and rise to 95% by 2020 of sexual acts protected by a condom among FSW and MSM; SC10) UNAIDS with condom drop in key populations: 90-90-90 objective reached in 2020 and 95-95-95 in 2030 and decline by 25 percentage point of sexual acts protected by a condom among FSW and MSM. (95% Credible Intervals are not presented to ease visual interpretation.)


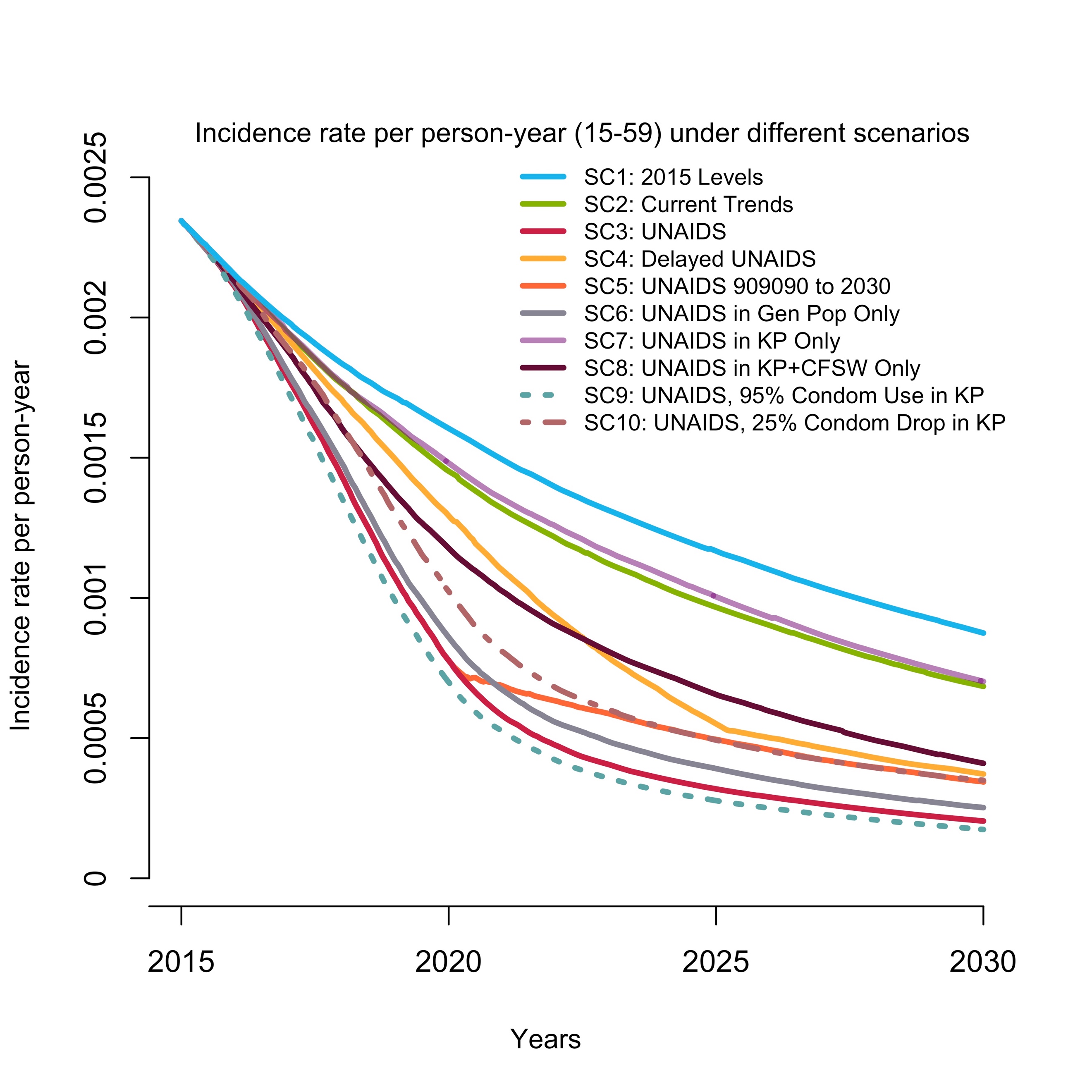


**Figure F. Predicted median HIV incidence per person-year among 15-59 years old in Côte d’Ivoire from 2015 to 2030 under different intervention coverage scenarios.** The scenarios are detailed in Table 1. SC1) Baseline: testing rate, antiretroviral (ART) recruitment rate, and ART failure rate stable at their 2015 values; SC2) Current trends: observed increase in those three rates from 2010-2015 projected through 2020; SC3) UNAIDS: 90-90-90 objective reached in 2020 and 95-95-95 in 2030; SC4) Delayed UNAIDS: 90-90-90 objective reached in 2025 and maintained to 2030; SC5) UNAIDS 909090 to 2030: 90-90-90 objective reached in 2020 and maintained to 2030, SC6) UNAIDS in general population: 90-90-90 objective reached in 2020 and 95-95-95 in 2025 among general population only; SC7) UNAIDS in key populations: 90-90-90 objective reached in 2020 and 95-95-95 in 2030 among FSW and MSM populations only; SC8) UNAIDS in key populations and CFSW: 90-90-90 objective reached in 2020 and 95-95-95 in 2030 among MSM, FSW, and CFSW only; SC9) UNAIDS plus condom in key populations: 90-90-90 objective reached in 2020 and 95-95-95 in 2030 and rise to 95% by 2020 of sexual acts protected by a condom among FSW and MSM; SC10) UNAIDS with condom drop in key populations: 90-90-90 objective reached in 2020 and 95-95-95 in 2030 and decline by 25 percentage point of sexual acts protected by a condom among FSW and MSM. (95% Credible Intervals are not presented to ease visual interpretation.)


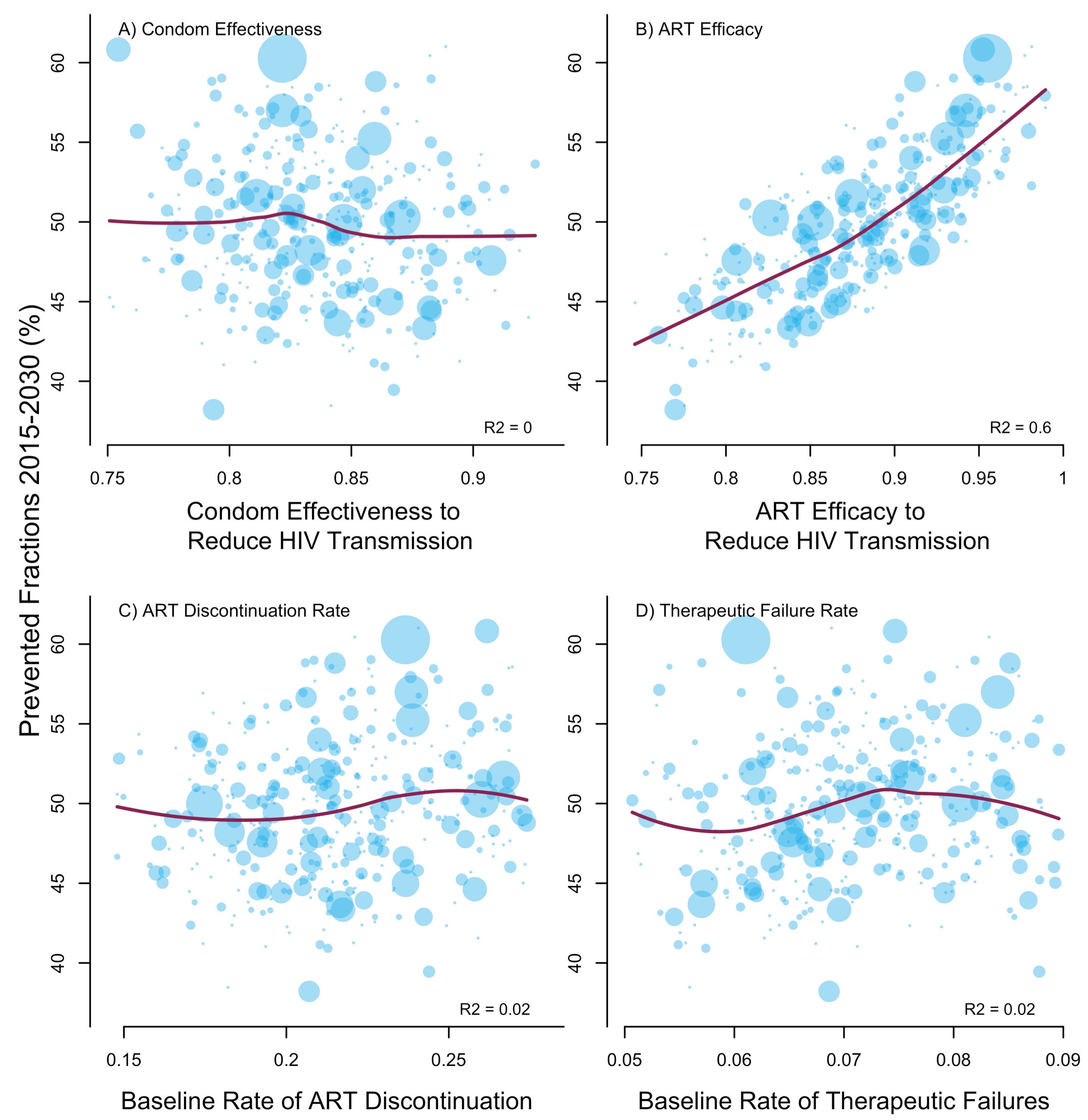


**Figure G**. **Sensitivity analysis of the impact of different model parameters on prevented fractions over 2015-2030 of the UNAIDS scenario (SC3), as compared to the baseline scenario (SC1).** The following parameters are presented: A) condom effectiveness, B) efficacy of antiretroviral therapy to reduce HIV transmission, C) baseline rate of ART discontinuation, and D) therapeutic failures. The four panels show the estimated prevented fractions as a function of the parameters’ posterior distribution. (Note: the size of the points is relative to their sampling importance weight.)


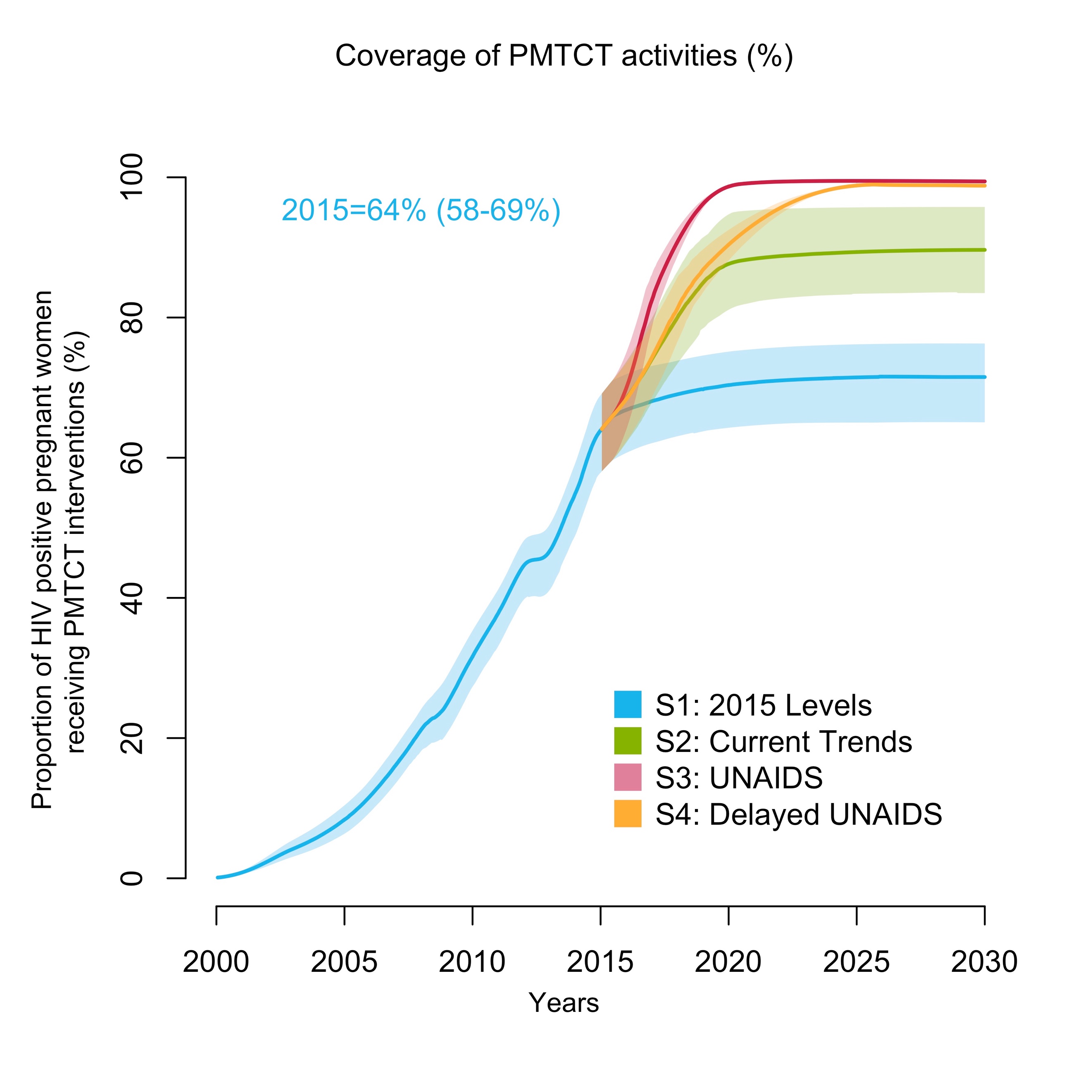


**Figure H. Coverage of prevention of mother-to-child transmission interventions under four intervention scenarios (median and 95% credible intervals).** The scenarios are detailed in Table 1. SC1) Baseline: testing rate, antiretroviral (ART) recruitment rate, and ART failure rate stable at their 2015 values; SC2) Current trends: observed increase in those three rates from 2010-2015 projected through 2020; SC3) UNAIDS: 90-90-90 objective reached in 2020 and 95-95-95 in 2030; SC4) Delayed UNAIDS: 90-90-90 objective reached in 2025 and maintained to 2030. (Note: Coverage is defined as receiving ART prophylaxis, ART treatment, or being already under ART treatment.)


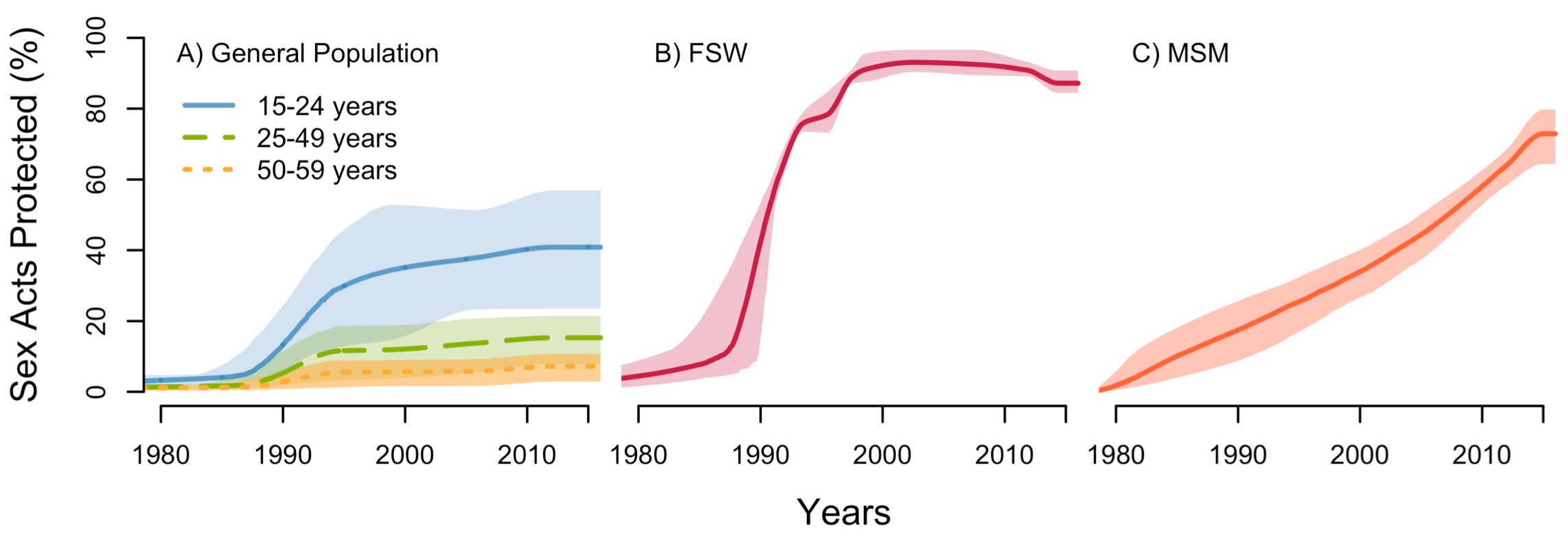


**Figure I**: Historical trends in condom use over 1980-2015 in Côte d’Ivoire for A) the general population (by age groups), B) female sex workers, and C) men who have sex with men. Median (lines) and 95% credible intervals (shaded areas) of the posterior distributions.

**References**

1. Maheu-Giroux M, Vesga J, Diabaté S, Alary M, Baral S, Diouf D, et al. Changing dynamics of HIV transmission in Côte d’Ivoire: modeling who acquired and transmitted infections and estimating the impact of past HIV interventions (1976-2015). JAIDS (Accepted). 2017.

2. UNDP. World Population Prospects:The 2015 Revision - Key Findings and Advance Tables. New York, NY: Population Division of the Department of Economic and Social Affairs of the United Nations Secretariat, 2015 Contract No.: ESA/P/WP.241.

3. Lewis JJ, Ronsmans C, Ezeh A, Gregson S. The population impact of HIV on fertility in sub-Saharan Africa. AIDS. 2004;18 Suppl 2:S35-43. PubMed PMID: 15319742.

4. N'Cho S, Kouassi L, Koffi A, Schoemaker J, Barrère M, Barrère B, et al. Enquête Démographique et de Santé, Côte d'Ivoire 1994. Calverton, MD: Institut National de la Statistique et Macro International Inc., 1995.

5. INS, ORC Macro. Enquête Démographique et de Santé, Côte d'Ivoire 1998-1999. Calverton, MD: Institute National de la Statistique et ORC Macro, 2001.

6. INS, MLS, ORC Macro. Enquête sur les Indicateurs du Sida, Côte d'Ivoire 2005. Calverton, MD: Institut National de la Statistique (INS), Ministère de la Lutte contre le Sida [Côte d'Ivoire] et ORC Macro., 2006.

7. INS, ICF International. Enquête Démographique et de Santé et à Indicateurs Multiples de Côte d'Ivoire 2011-2012. Calverton, MD: Institut National de la Statistique et ICF International, 2012.

8. MSLS. Étude sur le VIH et les facteurs de risques associés chez les hommes ayant des rapports sexuels avec des hommes à Abidjan, Côte d'Ivoire. Abidjan, Côte d'Ivoire: Ministère de la Santé et de la Lutte contre le VIH/SIDA. Programme de lutte contre le Sida en direction des populations hautement vulnérables., 2012.

9. UNAIDS. Combination HIV Prevention: Tailoring and Coordinating Biomedical, Behavioural and Structural Strategies to Reduce New HIV Infections. Geneva, Switzerland: Joint United Nations Programme on HIV/AIDS (UNAIDS), 2010.

10. Holland CE, Papworth E, Billong SC, Tamoufe U, LeBreton M, Kamla A, et al. Antiretroviral treatment coverage for men who have sex with men and female sex workers living with HIV in Cameroon. J Acquir Immune Defic Syndr. 2015;68 Suppl 2:S232-40. doi: 10.1097/QAI.0000000000000443. PubMed PMID: 25723989.

11. Hakim AJ, Aho J, Semde G, Diarrassouba M, Ehoussou K, Vuylsteke B, et al. The Epidemiology of HIV and Prevention Needs of Men Who Have Sex with Men in Abidjan, Cote d'Ivoire. PLoS One. 2015;10(4):e0125218. doi: 10.1371/journal.pone.0125218. PubMed PMID: 25909484.

12. Aho J, Hakim A, Vuylsteke B, Semde G, Gbais HG, Diarrassouba M, et al. Exploring risk behaviors and vulnerability for HIV among men who have sex with men in Abidjan, Cote d'Ivoire: poor knowledge, homophobia and sexual violence. PLoS One. 2014;9(6):e99591. doi: 10.1371/journal.pone.0099591. PubMed PMID: 24959923; PubMed Central PMCID: PMCPMC4069062.

13. Vandepitte J, Lyerla R, Dallabetta G, Crabbé F, Alary M, Buvé A. Estimates of the number of female sex workers in different regions of the world. Sex Transm Infect. 2006;82 Suppl 3:iii18-25. doi: 10.1136/sti.2006.020081. PubMed PMID: 16735288; PubMed Central PMCID: PMCPMC2576726.

14. Yao Kouamé M. Analyse situationnelle des interventions en IST/VIH/SIDA auprès des professionnel(le)s du sexe. Abidjan, Côte d'Ivoire: L'UNFPA Côte d'Ivoire et La Direction de coordination du Programme national de lutte contre le Sida chez les population hautement vulnérables, 2008.

15. Schwartz S, Papworth E, Thiam-Niangoin M, Abo K, Drame F, Diouf D, et al. An urgent need for integration of family planning services into HIV care: the high burden of unplanned pregnancy, termination of pregnancy, and limited contraception use among female sex workers in Côte d'Ivoire. J Acquir Immune Defic Syndr. 2015;68 Suppl 2:S91-8. doi: 10.1097/QAI.0000000000000448. PubMed PMID: 25723996.

16. Ghys PD, Diallo MO, Ettiègne-Traoré V, Kalé K, Tawil O, Caraël M, et al. Increase in condom use and decline in HIV and sexually transmitted diseases among female sex workers in Abidjan, Côte d'Ivoire, 1991-1998. AIDS. 2002;16(2):251-8. PubMed PMID: 11807310.

17. Bamba A, Grover E, Ezouatchi R, Thiam-Niangoin M, Papworth E, Grosso A, et al. Étude biologique et comportementale des IST/VIH/SIDA chez les professionnelles du sexe du district d'Abidjan et examen des interventions en direction des populations clefs en Côte d'Ivoire. Ministère de la Santé et de la Lutte contre le SIDA, ENDA Santé, Johns Hopkins University, 2014.

18. Lo Y, Sidibe C, Soro B, Kariburyo J. Prévention et prise en charge de l'infection du VHI/SIDA et des autres IST ciblant les professionnels du sexe et leurs partenaires en Côte d'Ivoire: rapport de la mission technique d'appui OMS. Geneva, Switzerland: Organisation mondiale de la Santé, 2009.

19. MSLS. Analyse des connaissances, attitudes et pratiques des professionnels(les) du sexe dans dix-huit villes de Côte d'Ivoire. Abidjan, Côte d'Ivoire: Ministère de la Santé et de la Lutte contre le Sida, 2012.

20. Vuylsteke BL, Ghys PD, Traoré M, Konan Y, Mah-Bi G, Maurice C, et al. HIV prevalence and risk behavior among clients of female sex workers in Abidjan, Côte d'Ivoire. AIDS. 2003;17(11):1691-4. doi: 10.1097/01.aids.0000060419.84040.61. PubMed PMID: 12853752.

21. Garnett GP, Anderson RM. Balancing sexual partnerships in an age and activity stratified model of HIV transmission in heterosexual populations. IMA J Math Appl Med Biol. 1994;11(3):161-92. PubMed PMID: 7822888.

22. Boily MC, Baggaley RF, Wang L, Masse B, White RG, Hayes RJ, et al. Heterosexual risk of HIV-1 infection per sexual act: systematic review and meta-analysis of observational studies. Lancet Infect Dis. 2009;9(2):118-29. doi: 10.1016/S1473-3099(09)70021-0. PubMed PMID: 19179227; PubMed Central PMCID: PMCPMC4467783.

23. Boily MC, Dimitrov D, Abdool Karim SS, Mâsse B. The future role of rectal and vaginal microbicides to prevent HIV infection in heterosexual populations: implications for product development and prevention. Sex Transm Infect. 2011;87(7):646-53. doi: 10.1136/sextrans-2011-050184. PubMed PMID: 22110117; PubMed Central PMCID: PMCPMC3332062.

24. Mackelprang RD, Baeten JM, Donnell D, Celum C, Farquhar C, de Bruyn G, et al. Quantifying ongoing HIV-1 exposure in HIV-1-serodiscordant couples to identify individuals with potential host resistance to HIV-1. J Infect Dis. 2012;206(8):1299-308. doi: 10.1093/infdis/jis480. PubMed PMID: 22926009; PubMed Central PMCID: PMCPMC3448964.

25. Naicker N, Kharsany AB, Werner L, van Loggerenberg F, Mlisana K, Garrett N, et al. Risk Factors for HIV Acquisition in High Risk Women in a Generalised Epidemic Setting. AIDS Behav. 2015;19(7):1305-16. doi: 10.1007/s10461-015-1002-5. PubMed PMID: 25662962; PubMed Central PMCID: PMCPMC4506252.

26. Cohen MS, Chen YQ, McCauley M, Gamble T, Hosseinipour MC, Kumarasamy N, et al. Prevention of HIV-1 infection with early antiretroviral therapy. N Engl J Med. 2011;365(6):493-505. doi: 10.1056/NEJMoa1105243. PubMed PMID: 21767103; PubMed Central PMCID: PMCPMC3200068.

27. Weller S, Davis K. Condom effectiveness in reducing heterosexual HIV transmission. Cochrane Database Syst Rev. 2002;(1):CD003255. doi: 10.1002/14651858.CD003255. PubMed PMID: 11869658.

28. Hollingsworth TD, Anderson RM, Fraser C. HIV-1 transmission, by stage of infection. J Infect Dis. 2008;198(5):687-93. doi: 10.1086/590501. PubMed PMID: 18662132.

29. Wandel S, Egger M, Rangsin R, Nelson KE, Costello C, Lewden C, et al. Duration from seroconversion to eligibility for antiretroviral therapy and from ART eligibility to death in adult HIV-infected patients from low and middle-income countries: collaborative analysis of prospective studies. Sex Transm Infect. 2008;84 Suppl 1:i31-i6. doi: 10.1136/sti.2008.029793. PubMed PMID: 18647863; PubMed Central PMCID: PMCPMC2569418.

30. Vo TT, Ledergerber B, Keiser O, Hirschel B, Furrer H, Battegay M, et al. Durability and outcome of initial antiretroviral treatments received during 2000--2005 by patients in the Swiss HIV Cohort Study. J Infect Dis. 2008;197(12):1685-94. doi: 10.1086/588141. PubMed PMID: 18513155.

31. Granich RM, Gilks CF, Dye C, De Cock KM, Williams BG. Universal voluntary HIV testing with immediate antiretroviral therapy as a strategy for elimination of HIV transmission: a mathematical model. Lancet. 2009;373(9657):48-57. doi: 10.1016/S0140-6736(08)61697-9. PubMed PMID: 19038438.

32. Ray M, Logan R, Sterne JA, Hernández-Díaz S, Robins JM, Sabin C, et al. The effect of combined antiretroviral therapy on the overall mortality of HIV-infected individuals. AIDS. 2010;24(1):123-37. doi: 10.1097/QAD.0b013e3283324283. PubMed PMID: 19770621; PubMed Central PMCID: PMCPMC2920287.

33. Estill J, Ford N, Salazar-Vizcaya L, Haas AD, Blaser N, Habiyambere V, et al. The need for second-line antiretroviral therapy in adults in sub-Saharan Africa up to 2030: a mathematical modelling study. Lancet HIV. 2016;3(3):e132-9. doi: 10.1016/S2352-3018(16)00016-3. PubMed PMID: 26939736.

34. Estill J, Aubrière C, Egger M, Johnson L, Wood R, Garone D, et al. Viral load monitoring of antiretroviral therapy, cohort viral load and HIV transmission in Southern Africa: a mathematical modelling analysis. AIDS. 2012;26(11):1403-13. doi: 10.1097/QAD.0b013e3283536988. PubMed PMID: 22421243; PubMed Central PMCID: PMCPMC3750130.

35. DIPE. Rapport annuel VIH/Sida du secteur santé en Côte d'Ivoire 2007-2008. Abidjan, Côte d'Ivoire: Direction de l'Information, de la Planification et de l'Évaluation. Ministère de la Santé et de l'Hygiène Publique, 2009.

36. DIPE. Rapport annuel des indicateurs VIH du secteur santé en Côte d'Ivoire 2009. Abidjan, Côte d'Ivoire: Direction de l'Information, de la Planification et de l'Évaluation. Ministère de la Santé et de l'Hygiène Publique., 2010.

37. DIPE. Rapport annuel des indicateurs VIH du secteur Santé en Côte d'Ivoire 2010. Abidjan, Côte d'Ivoire: Direction de l'Information, de la Planification et de l'Évaluation. Ministère de la Santé et de l'Hygiène Publique., 2011.

38. DIPE. Rapport annuel des indicateurs VIH du secteur santé en Côte d'Ivoire 2011. Abidjan, Côte d'Ivoire: Direction de l'Information, de la Planification, et de l'Évaluation. Ministère de la Santé et de la Lutte contre le Sida, 2012.

39. DIPE. Rapport annuel des indicateurs VIH du secteur santé en Côte d'Ivoire 2012. Abidjan, Côte d'Ivoire: Direction de l'Information, de la Planification et de l'Évaluation. Ministère de la Santé et de la Lutte contre le SIDA., 2013.

40. DIPE. Rapport annuel des indicateurs VIH du secteur santé en Côte d'Ivoire 2013. Abidjan, Côte d'Ivoire: Direction de l'Information, de la Planification et de l'Évaluation, 2014.

41. Vuylsteke B, Semdé G, Auld AF, Sabatier J, Kouakou J, Ettiègne-Traoré V, et al. Retention and risk factors for loss to follow-up of female and male sex workers on antiretroviral treatment in Ivory Coast: a retrospective cohort analysis. J Acquir Immune Defic Syndr. 2015;68 Suppl 2:S99-S106. doi: 10.1097/QAI.0000000000000442. PubMed PMID: 25723997.

42. Rollins N, Mahy M, Becquet R, Kuhn L, Creek T, Mofenson L. Estimates of peripartum and postnatal mother-to-child transmission probabilities of HIV for use in Spectrum and other population-based models. Sex Transm Infect. 2012;88 Suppl 2:i44-51. doi: 10.1136/sextrans-2012-050709. PubMed PMID: 23172345; PubMed Central PMCID: PMCPMC3512432.

43. MEF. Enquête Ivoirienne sur la fécondité 1980-81 - Rapport Principal. Volume I. Abidjan, Côte d'Ivoire: Ministère de l'Économie et des Finances. Direction de la Statistique, 1984.

44. MEF. Enquête Ivoirienne sur la fécondité 1980-81 - Rapport Principal. Volume II. Abidjan, Côte d'Ivoire: Ministère de l'Économie et des Finances. Direction de la Statistique., 1984.

45. Wade A, Kane C, Diallo P, Diop A, Gueye K, Mboup S, et al. HIV infection and sexually transmitted infections among men who have sex with men in Senegal. Aids. 2005;19(18):2133-40. doi: 10.1097/01.aids.0000194128.97640.07. PubMed PMID: WOS:000233493200010.

46. UNICEF. Enquête à Indicateurs Mulitples MICS2000 - Côte d'Ivoire. Abidjan, Côte d'Ivoire: UNICEF, 2000.

47. MLS. Stratégie nationale de communication pour le changement de comportement face au VIH/SIDA 2005-2008. Abidjan, Côte d'Ivoire: Ministère de la Lutte contre le SIDA, 2005.

48. DIPE. Rapport annuel des indicateurs VIH du secteur santé en Côte d'Ivoire 2014 - Non consolidé. Abidjan, Côte d'Ivoire: Direction de l'Information, de la Planification et de l'Évaluation. Ministère de la santé et de la lutte contre le sida., 2015.

49. Benoit SN, Gershy-Damet GM, Coulibaly A, Koffi K, Sangare VS, Koffi D, et al. Seroprevalence of HIV infection in the general population of the Côte d'Ivoire, West Africa. J Acquir Immune Defic Syndr. 1990;3(12):1193-6. PubMed PMID: 2173745.
