# Supplementary material for: Population-level impact of an accelerated HIV response plan to reach the UNAIDS 90-90-90 target in Côte d’Ivoire: Insights from mathematical modeling
Source: PLoS Med. 2017 Jun 15;14(6):e1002321. doi: 10.1371/journal.pmed.1002321 (PMC5472267; doi:10.1371/journal.pmed.1002321)
Supplement: S2 Appendix — (DOCX) [file pmed.1002321.s002.docx]

**Modélisation de l’impact populationnel de l’accélération de la riposte au VIH pour atteindre la cible 90-90-90 d’ONUSIDA en Côte d’Ivoire**

Mathieu Maheu-Giroux, Juan F Vesga, Souleymane Diabaté, Michel Alary, Stefan Baral, Daouda Diouf, Kouamé Abo, and Marie-Claude Boily

**Résumé**

**Introduction** : Les programmes de lutte contre le VIH devront être rapidement mis à l’échelle pour atteindre les ambitieux objectifs proposés par ONUSIDA. Ces derniers visent à ce que 90% des personnes vivant avec le VIH connaissent leur statut, que 90% de ces derniers reçoivent des antirétroviraux et que 90% des personnes sous traitement aient une charge virale indétectable d’ici 2020 (et 95% pour chacun de ces indicateurs d’ici 2030). Notre étude vise à estimer l’impact de plusieurs scénarios de traitement comme prévention en Côte d’Ivoire, un des pays d’Afrique de l’Ouest avec une incidence élevée et où les populations clés influencent la dynamique de transmission.

**Méthodes et résultats**: Un modèle mathématique dynamique de type déterministe stratifié par groupes d’âge a été développé et calibré, dans un cadre Bayésien, aux données épidémiologiques et programmatiques du pays. Le modèle prend en compte la transmission sexuelle et verticale du VIH dans la population générale, chez les travailleuses du sexe (TS) et les hommes ayant des rapports sexuels avec des hommes (HSH). Nous avons évalué l’impact d’une accélération de la riposte pour atteindre les cibles ONUSIDA, et celles de huit autres scénarios, sur la transmission du VIH chez les adultes et les enfants, comparativement à un scénario de base. Ce scénario de base correspond aux taux atteints par le pays en 2015 en termes de dépistage, d’initiation de traitement antirétroviral, d’interruption de traitement, d’échecs thérapeutiques et de proportion d’actes sexuels protégés par un condom. Pour l’année de référence 2015, nous avons estimé avec le modèle que 52% (intervalle de crédibilité à 95% : 46-58%) des personnes vivant avec le VIH connaissaient leur statut, que 72% (57-82%) des personnes diagnostiquées recevaient un traitement antirétroviral et que 77% (74-79%) des personnes recevant des antirétroviraux avaient une charge virale indétectable. Atteindre les cibles ONUSIDA à temps permettrait de prévenir 50% (42-60%) des nouveaux cas de VIH sur la période 2015-2030, comparativement à 30% (25-36%) si l’objectif 90-90-90 était atteint avec cinq années de retard, en 2025. Si la cible ONUSIDA est atteinte chez les TS, leurs clients, et les HSH – mais pas dans le reste de la population – une fraction similaire de nouvelles infections (30%; 21-39%) sera prévenue. Une diminution absolue de 25% des rapports sexuels protégés par un condom chez les TS et les HSH, comparativement à 2015, réduirait l’impact de la cible ONUSIDA à seulement 38% (26-51%) d’infections prévenues. Une limitation de cette étude réside dans la modélisation de l’épidémie au niveau national avec le postulat que les niveaux de couverture des interventions sont géographiquement homogènes. Les recherches futures devraient examiner si la prise en compte des taux de couverture des interventions spécifiques à chaque région affecterait la dynamique de transmission du VIH.

**Conclusions**: Pour maximiser l’impact populationnel de l’objectif ONUSIDA, il faudrait accélérer de manière significative la riposte au VIH, particulièrement en ce qui a trait aux activités de dépistage, d’initiation et d’interruption du traitement. Cibler les populations clés et les clients des TS pourrait réduire la transmission de façon efficiente. Maintenir à leur haut niveau la proportion de rapport sexuels protégés par un condom chez les populations clés doit demeurer un important pilier des programmes de prévention.
